# Core microbes in *Cordyceps militaris* sclerotia and their nitrogen metabolism-related ecological functions

**DOI:** 10.1128/spectrum.01053-24

**Published:** 2024-08-20

**Authors:** Li Luo, Fei Dai, Zhongshun Xu, Jingqiang Guan, Gangxiang Fei, Jiaojiao Qu, Min Yao, Yuan Xue, Yeming Zhou, Xiao Zou

**Affiliations:** 1 Institute of Fungus Resources, College of Life Science, Guizhou University, Guiyang, Guizhou, China; 2 Anshun Branch of Guizhou Tobacco Company, Anshun, Guizhou, China; 3 Key Laboratory of Plant Resource Conservation and Germplasm Innovation in Mountainous Region, Guizhou University, Guiyang, Guizhou, China; Broad Institute, Cambridge, Massachusetts, USA

**Keywords:** *Cordyceps militaris*, core microbiome, microbial function, nitrogen metabolism, community assembly, cordycepin

## Abstract

*Cordyceps militaris* infects insects and forms sclerotia within the insect remains, establishing insect–microbe complexes. Here, *C*. *militaris* sclerotia samples from a single location in China over a 5-year period were subjected to high-throughput DNA sequencing, and the core microbes (which were stably enriched in the sclerotia over the 5 years) were identified. Next, seven bacterial strains were isolated from the *C. militaris* sclerotia, their biochemical characteristics were assessed, and they were co-cultured with *C. militaris* to study their effects on *C. militaris* metabolite production and biomass. Furthermore, the effects of NH_4_, NO_3_, and peptone media on *C. militaris* were compared. The results showed that *Rhodococcus*, *Phyllobacterium*, *Pseudomonas*, *Achromobacter*, *Ensifer*, *Stenotrophomonas*, *Sphingobacterium*, *Variovorax*, and *Acinetobacter* were the core microbes. Although co-culture of *C. militaris* with the seven bacterial strains isolated from the sclerotia did not directly increase the cordycepin level, they all had NO_3_ reduction ability, and four had urea decomposition ability. Meanwhile, *C. militaris* in NH_4_ medium had an increased cordycepin level compared to *C. militaris* in the other two media. From this, we inferred that bacteria in the sclerotia can convert NO_3_ to NH_4_, and then cordycepin is produced using NH_4_, which was confirmed by RNA-seq and real-time fluorescence quantitative PCR. Thus, bacteria in the sclerotia may indirectly affect the *C. militaris* metabolite production by regulating nitrogen metabolism. In summary, there are stable core microbes in the *C. militaris* sclerotia, and they may directly and indirectly affect the growth and metabolite production of *C. militaris*.

**IMPORTANCE:**

The model *Cordyceps* species *Cordyceps militaris* is rich in therapeutic compounds. It has recently been demonstrated that symbiotic microbes in sclerotia affect *Cordyceps’* growth, development, and secondary metabolite production. In this study, core microbes were identified based on *C. militaris* sclerotia samples obtained from the same site over 5 years. Additionally, bacterial strains isolated from *C. militaris* sclerotia were found to affect metabolite production and nitrogen utilization, based on functional tests. Moreover, based on the bacterial nitrogen metabolism capacity in the sclerotia and its influence on *C. militaris* metabolite production, we deduced that bacteria in the sclerotia can indirectly affect *C. militaris* metabolite production by regulating nitrogen metabolism. This is the first report on how bacteria in the sclerotia affect *C. militaris* metabolite production from the perspective of the nitrogen cycle. The results increase our understanding of microbial functions in *C. militaris* sclerotia.

## INTRODUCTION

*Cordyceps militaris* is an important medicinal fungus (1). It produces a variety of active substances, including cordycepin, cordyceps polysaccharide, cordycepic acid, and adenosine (2, 3). These substances have been found to improve human immunity, combat fatigue, inhibit tumor growth, lower blood sugar levels, reduce inflammation, and decrease uric acid levels (4–7). They may also be useful for treating COVID-19 and major depressive disorder (8, 9).

*C. militaris* infects insects and forms sclerotia within the insect remains, establishing insect–microbe complexes. Thus, there are many microbes in the insect body. There have been several reports on the microbial diversity (10–12), and the microbial composition affects the function of the community (13, 14).

In this study, first, we identified the stable core microbes in *C. militaris* sclerotia. Studies have mostly sampled from multiple sites rather than from one site over many years, but exploring the stability of microbes over time is valuable. As a stable presence is required for a microbe to function as a core microbe, we explored the microbial stability, then considered microbial enrichment in sclerotia, and then synthesized both sets of results to identify the core microbes. Second, we explored the effects of these core microbes on *C. militaris*. Microbes are known to affect *Cordyceps sinensis* growth, development, and infection (13); *Herbaspirillum* and *Phyllobacterium* are known to increase bioactive compounds in *C. militaris* (11), and *Strophomonas maltophilia* and *Pseudomonas baetica* are known to affect the *C. militaris* biomass, cordycepin level, and polysaccharide level (10). However, the effects of other bacteria are less known. Therefore, it was necessary to isolate further bacteria and conduct experimental testing. Third, we explored whether nitrogen conversion by the microbes in sclerotia can influence *C. militaris* metabolite production. Cordycepin, carotenoid, and superoxide dismutase levels have been reported to be higher in artificially cultivated *Cordyceps* fruiting bodies on pupae than those on wheat (14). As sclerotia are rich in nutrients, such as protein (>60%) and fat (>15%) (15), they may promote fruiting bodies growth (16), and as there are many microbes in the sclerotia (10–12), they represent a very metabolically active site (17). We speculated that in addition to the nutrients in the sclerotia directly affecting *C. militaris* metabolite production (18), the microbes in the sclerotia may also influence it. *Mesorhizobium*, *Achromobacter*, *Pantobacter*, and *Stenotrophomonas* in *C. militaris* sclerotia can metabolize urea and reduce NO_3_ (10), while NH_4_ medium can increase the extracellular polysaccharide level of *Cordyceps sinensis* (19). By exploring whether microbial nitrogen conversion in sclerotia can influence the effect of nitrogen on *Cordycep*s metabolite production, a coherent functional picture was formed, expanding our understanding of the microbial functions in *C. militaris* in sclerotia.

In this study, we analyzed *C. militaris* sclerotia samples collected from a single site in Liaoning Province, China, over 5 years to identify the stable core microbes, verify the nitrogen metabolism functions of the isolated bacterial strains, and co-culture these bacterial strains with *C. militaris*. RNA-seq and real-time fluorescence quantitative PCR (RT-qPCR) were employed to assess the impacts of the isolated bacterial strains and NH_4_ and NO_3_ media on *C. militaris* metabolite production. Finally, based on the bacterial nitrogen metabolism capacity in the sclerotia and its influence on *C. militaris* metabolite production, we deduced that bacteria in the sclerotia affect cordycepin production indirectly by regulating nitrogen metabolism. The results deepen our understanding of microbial ecological functions and provide a way to increase the cordycepin yield.

## RESULTS

### Core microbes in *C. militaris* sclerotia

**Fig 1:**
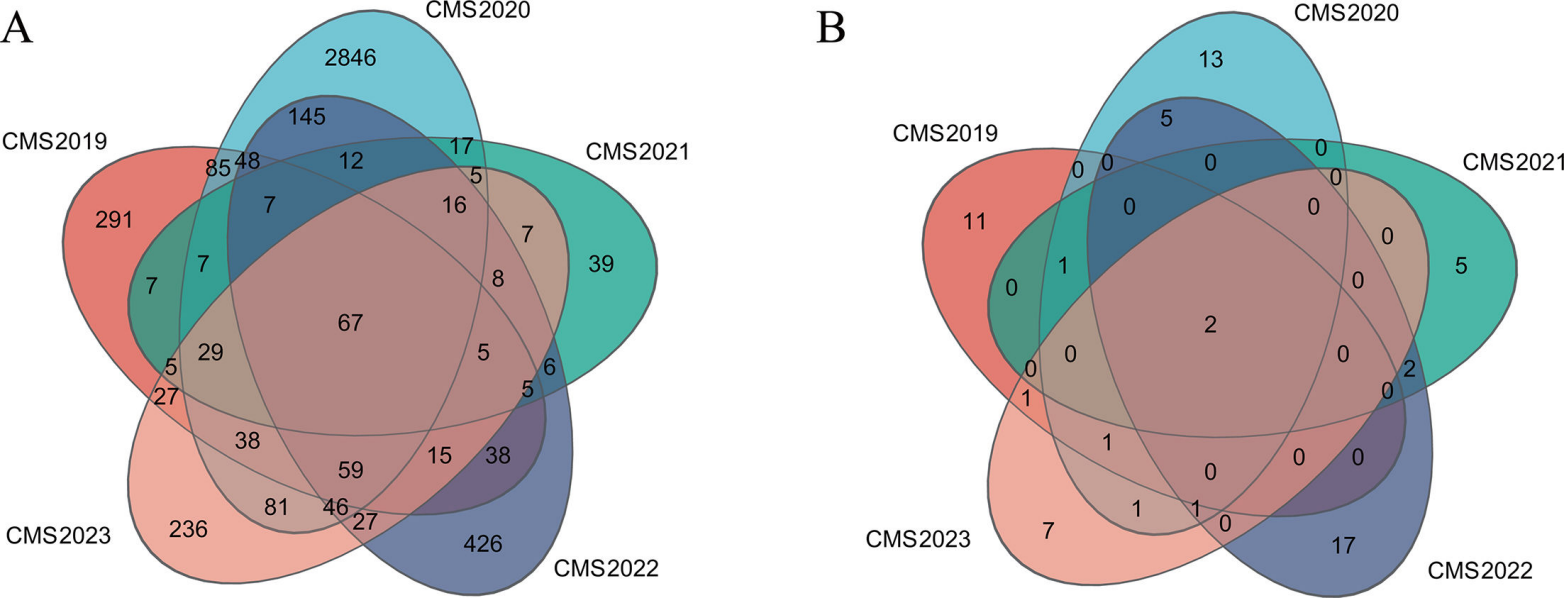
Venn diagrams of the OTUs in *C. militaris* sclerotia across 5 years. (**A**) Bacterial and (**B**) fungal OTUs. “CMSyear” indicates *C. militaris* sclerotia samples from a year between 2019 and 2023.

**Fig 2:**
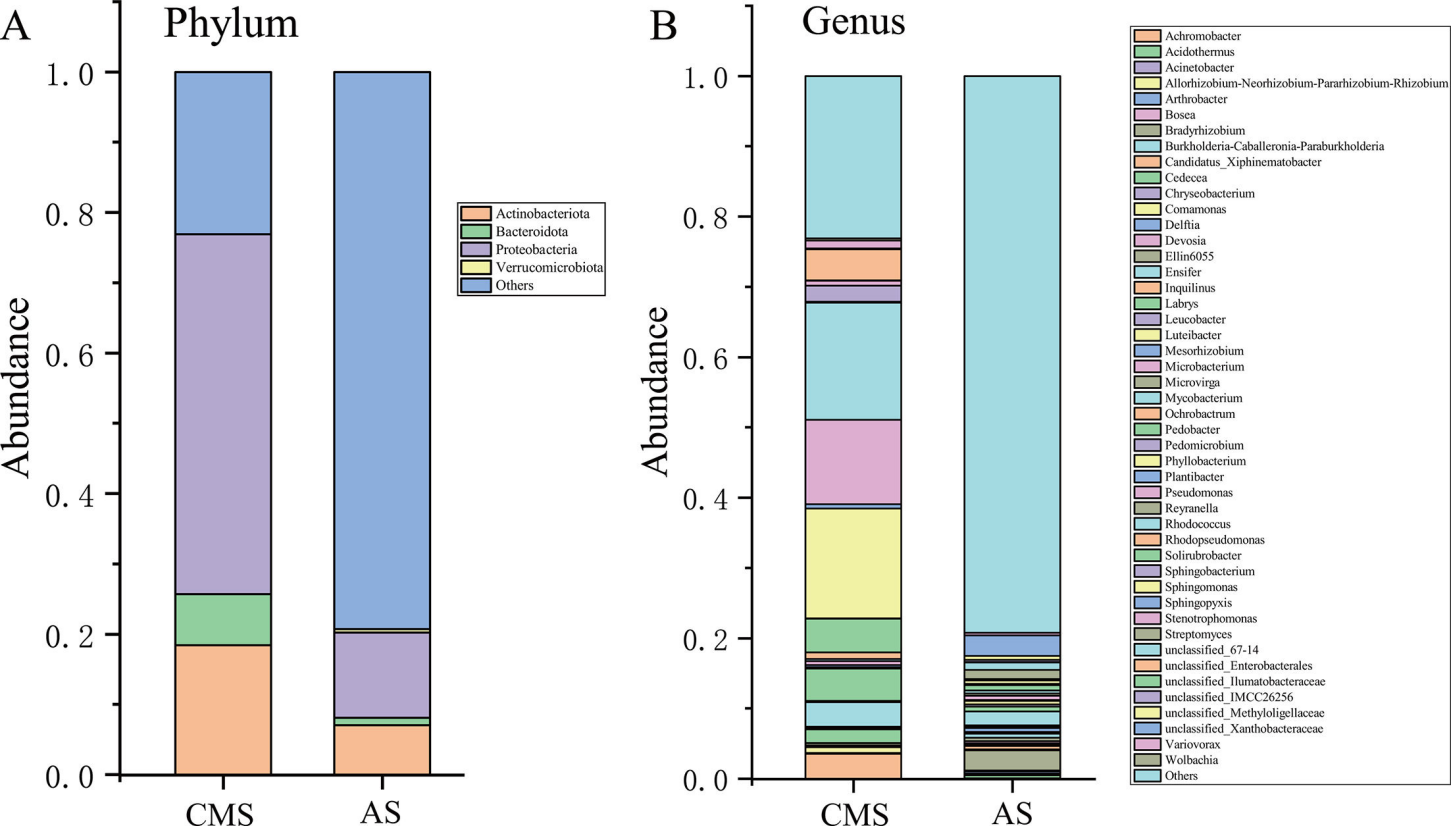
Relative abundances of 67 bacterial OTUs in *C. militaris* sclerotia and attached soil. (**A**) Phylum and (**B**) genus level. CMS, *C. militaris* sclerotia samples. AS, attached soil 1 cm around the *C. militaris*.

The high-throughput sequencing data from *C. milit*aris sclerotia obtained from a single site showed that 67 bacterial operational taxonomic units (OTUs) were consistently present over the 5 years assessed (Fig. 1). The 67 OTUs belonged to four phyla, namely, Proteobacteria (51.21%), Actinobacteriota (18.41%), Bacteroidota (7.26%), and Verrucomicrobiota (0.01%) (Fig. 2A; Table S1). The 67 OTUs belonged to 47 genera.

We defined “core microbes” as genera with a relative abundance >0.1% that were enriched in the sclerotia relative to the attached soil. Thus, there were at least nine core microbial genera, which included *Rhodococcus* (sclerotia: 16.69%, attached soil: 0.44%), *Phyllobacterium* (sclerotia: 15.65%, attached soil: 0.53%), *Pseudomonas* (sclerotia: 11.97%, attached soil: 0.61%), *Achromobacter* (sclerotia: 3.57%, attached soil: 0.08%), *Ensifer* (sclerotia: 3.51%, attached soil: 0.53%), *Stenotrophomonas* (sclerotia: 2.48%, attached soil: 0.14%), *Sphingobacterium* (sclerotia: 2.3%, attached soil: 0.16%), *Variovorax* (sclerotia: 1.13%, attached soil: 0.32%), and *Acinetobacter* (sclerotia: 0.33%, attached soil: 0.00%) (Fig. 2B; Table S1).

The correlations among the 67 bacterial OTUs were 94.92% positive and 5.08% negative in the sclerotia, and 86.29% positive and 13.71% negative in the attached soil (Fig. S1). This larger proportion of positive correlations (indicating reciprocity rather than competition) in sclerotia compared to attached soil indicates that the relationships among the OTUs were more reciprocal.

There were only two fungal OTUs that were consistently present over the 5 years assessed (Fig. 1B); these were *C. militaris*, indicating that *C. militaris* was the dominant fungus in the sclerotia, and the impact of other fungi was negligible. Therefore, in this paper, the microbial diversity and core microbes are primarily discussed in terms of bacteria.

### Isolation and identification of bacterial strains

After discarding duplicate bacterial species (using molecular methods), there were seven bacterial strains isolated from the sclerotia. They were identified using bacterial biochemical identification strips (Table S2) and Basic Local Alignment Search Tool (BLAST) analyses of the 16S rRNA and *gyrB* gene sequences. After each genus was determined by the BLAST analysis, we selected sequences of strains from different species belonging to the same genus to construct phylogenetic trees of these seven strains (Fig. S2).

Strain E49 was nearly spherical (0.94–1.12 × 0.85–0.89 µm) and gram negative; colonies were round, off-white, with regular edges, and a smooth, opaque surface. Physiological and biochemical tests (Table S2) indicated that the strain was not athletic (lacked vigor), capable of amino acid decomposition to produce amines and CO_2_, alkalinized the medium, did not decompose urea, and did not decompose glucose to produce pyruvate. In addition, it could not utilize mannitol, inositol, sorbitol, or maltose as carbon sources. Using the methods outlined in *Bergey’s Manual of Systematic Bacteriology* (Eighth Edition), strain E49 was identified as *Variovorax gossypii*. According to the 16S rRNA gene sequence analysis, strain E49 shared 99% identity with *Variovorax gossypii* JM-310 (NR 178837). The *gyrB* gene sequence analysis led to a similar result as that of the 16 s rRNA gene sequence analysis. Thus, strain E49 was identified as *Variovorax gossypii*.

Using the same methods, strains E28, J16, B21, B26, D4, and C1 were identified as *Rhodococcus jostii*, *Achromobacter marplatensis*, *Acinetobacter lwoffii*, *Sphingobacterium multivorum*, *Mycobacterium stephanolepidis*, and *Pseudomonas protegens*, respectively (Table S2; Fig. S2).

### Nitrogen metabolism of bacteria in *C. militaris* sclerotia

**Fig 3:**
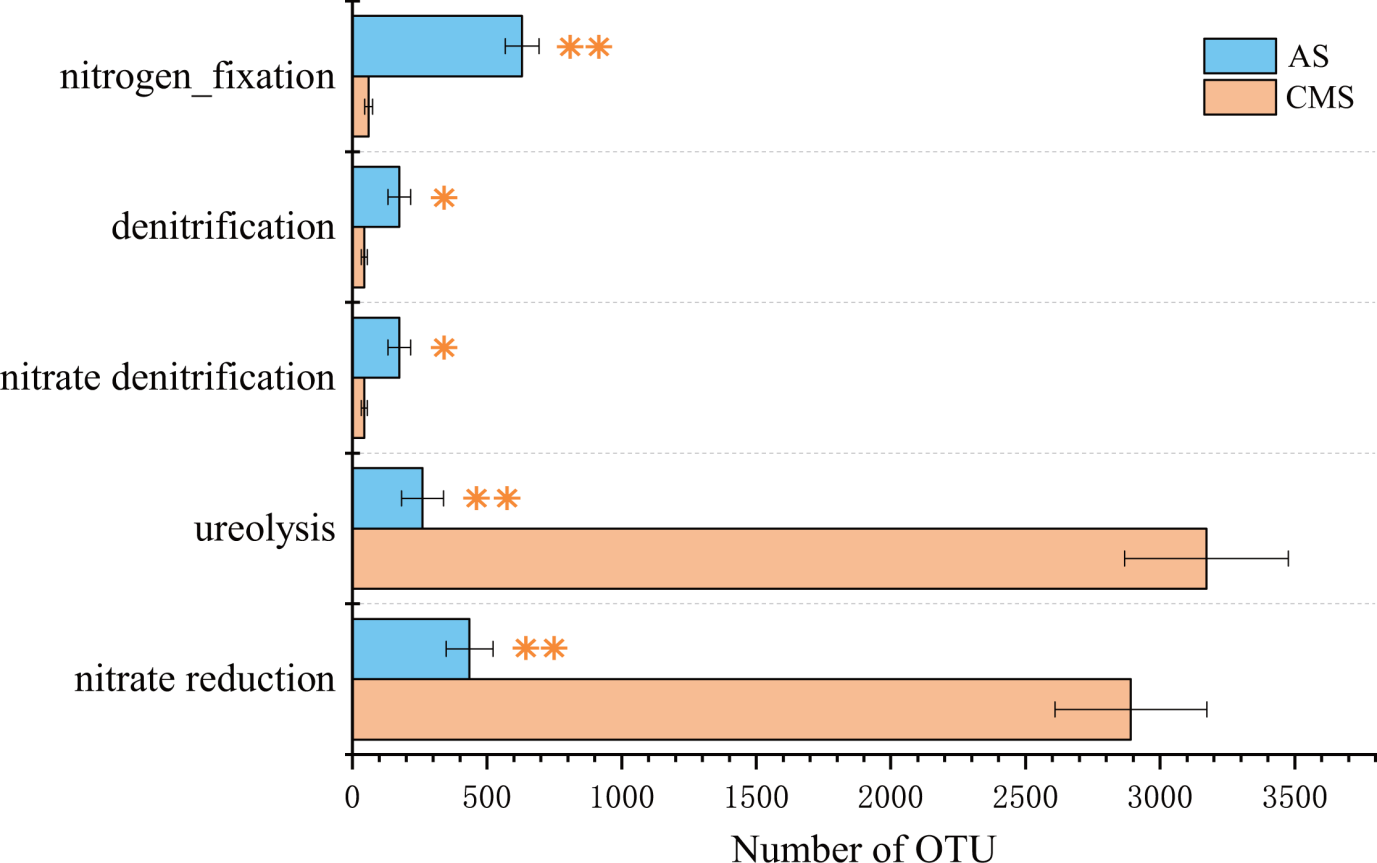
Functions of microbial community of *C. militaris* sclerotia and attached soil based on FAPROTAX predictions. *, *P* < 0.05; **, *P <* 0.01.

According to the Functional Annotation of Prokaryotic Taxa (FAPROTAX) prediction results, the abundance of OTUs related to NO_3_ reduction and the abundance of OTUs related to urea decomposition in the *C. militaris* sclerotia were significantly higher than the abundance of OTUs related to other types of nitrogen metabolism (*P* < 0.01) (Fig. 3). Additionally, the abundance of OTUs related to NO_3_ reduction and the abundance of OTUs related to urea decomposition were significantly higher in the sclerotia than the attached soil (*P* < 0.01). In contrast, the abundance of OTUs related to denitrification and the abundance of OTUs related to nitrogen fixation were higher in the attached soil than the sclerotia (*P* < 0.05, P < 0.01).

All seven isolated bacterial strains had the capacity for NO_3_ reduction (Table 1), and the relative abundance of these seven strains (among the 67 OTUs) was 31.60% in the sclerotia and 6.65% in the attached soil (Table S1). Four of the seven bacterial strains had the capacity for urea decomposition (Table 1), and the relative abundance of these four strains (among the 67 OTUs) was 15.50% in the sclerotia and 4.87% in the attached soil (Table S1). In other words, the nitrogen metabolism capacity (in terms of NO_3_ reduction capacity and urea decomposition capacity) of the bacteria in the sclerotia was higher than that in the attached soil, which helps to validate the FAPROTAX prediction results on the microbial community in the sclerotia (Fig. 3).

**TABLE 1:** Functions of seven bacterial strains isolated from *C. militaris* sclerotia^*a*^

Strain	NO_3_ reduction	Urea lysis
*Rhodococcus jostii* E28	＋	−
*Achromobacter marplatensis* J16	＋	−
*Acinetobacter lwoffii* B21	＋	＋
*Sphingobacterium multivorum* B26	＋	＋
*Mycobacterium stephanolepidis* D4	＋	＋
*Variovorax gossypii* E49	＋	−
*Pseudomonas protegens* C1	＋	＋

^*a*^ “+,” positive reaction; “−,” nonexistent reaction.

### Effects of different nitrogen sources on *C. militaris*

**Fig 4:**
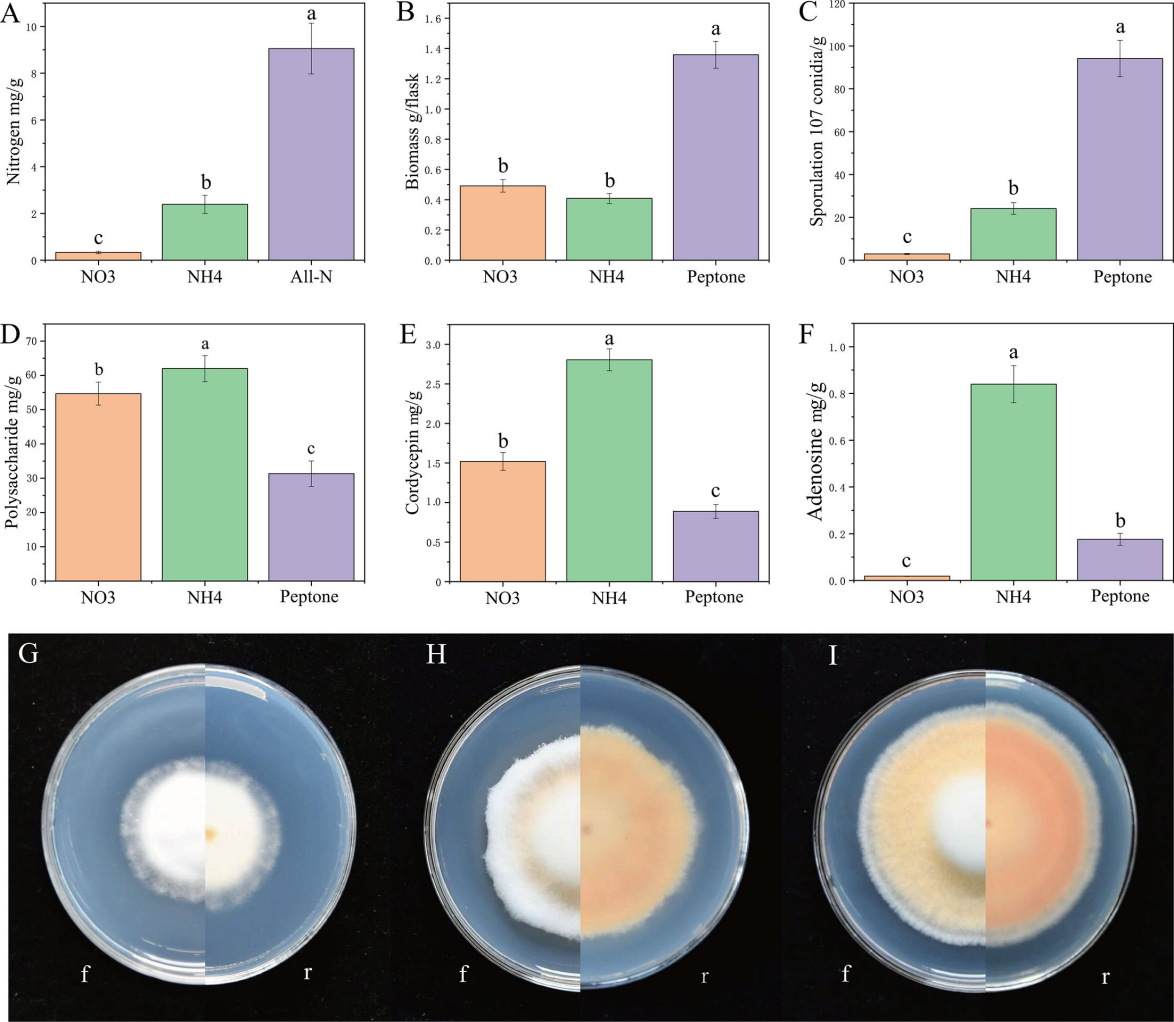
Effects of nitrogen sources on *C. militaris*. (**A**) Levels of various nitrogen sources in *C. militaris* sclerotia. (**B**) Mycelium biomass, (**C**) sporulation, (**D**) extracellular polysaccharide level, (**E**) cordycepin level, and (**F**) adenosine level in media with different nitrogen sources. Values are mean ± SD from three independent experiments. Different letters above the bars indicate significant differences (*P* < 0.05). *C. militaris* growth on different nitrogen sources for 21 days: (**G**) NO_3_, (**H**) NH_4_, and (**I**) peptone. f, front; r, reverse.

In the *C. militaris* sclerotia, the NH_4_–N level was significantly higher than the NO_3_–N level (*P* < 0.01) (Fig. 4A). Furthermore, the combined quantity of these two forms of nitrogen was notably different from the total nitrogen level, suggesting the presence of other nitrogen compounds (Fig. 4A).

The mycelium biomass was not significantly different in the NH_4_ vs NO_3_ medium (*P* = 0.135) but significantly lower in the NH_4_ or NO_3_ vs the peptone medium (*P* < 0.01) (Fig. 4B). This indicates that *C. militaris* can utilize inorganic nitrogen, but its growth was much slower than its growth with organic nitrogen. The *C. militaria* spore yield was significantly higher in the peptone vs NH_4_ medium (*P* < 0.01) and the NH_4_ vs NO_3_ medium (*P* < 0.01) (Fig. 4C). The polysaccharide level was not significantly different in the NH_4_ vs NO_3_ medium (*P* = 0.06) but significantly higher in the NH_4_ or NO_3_ medium vs peptone medium (*P* < 0.01) (Fig. 4D). The cordycepin level was significantly higher in both the NH_4_ or NO_3_ vs peptone medium (*P* < 0.01), and the NH_4_ vs NO_3_ medium (*P* = 0.01) (Fig. 4E). The adenosine level was significantly higher in the NH_4_ vs peptone medium (*P* < 0.01) and the peptone vs NO_3_ medium (*P* < 0.01) (Fig. 4F).

On NH_4_ agar medium, the mycelia were fluffier; they were somewhat yellow/orange on the front side of the plate and more orange on the reverse side (Fig. 4H). In contrast, on the NO_3_ agar medium, the mycelia were firmer and off-white (Fig. 4G). Based on the absorbance at 445 nm, *C. militaris* produced more carotenoids on NH_4_ vs NO_3_ agar medium (Fig. S3) and peptone vs NO_3_ or NH_4_ agar medium (Fig. S3). This suggests that organic nitrogen was more favorable than inorganic nitrogen for carotenoid formation.

### RNA-seq analysis of *C. militaris* cultured in media with different nitrogen sources

There were 763 upregulated and 517 downregulated differentially expressed genes (DEGs) in the NH_4_ vs peptone medium (Fig. S4), and 681 upregulated and 630 downregulated DEGs in the NO_3_ vs peptone medium (Fig. S4).

**Fig 5:**
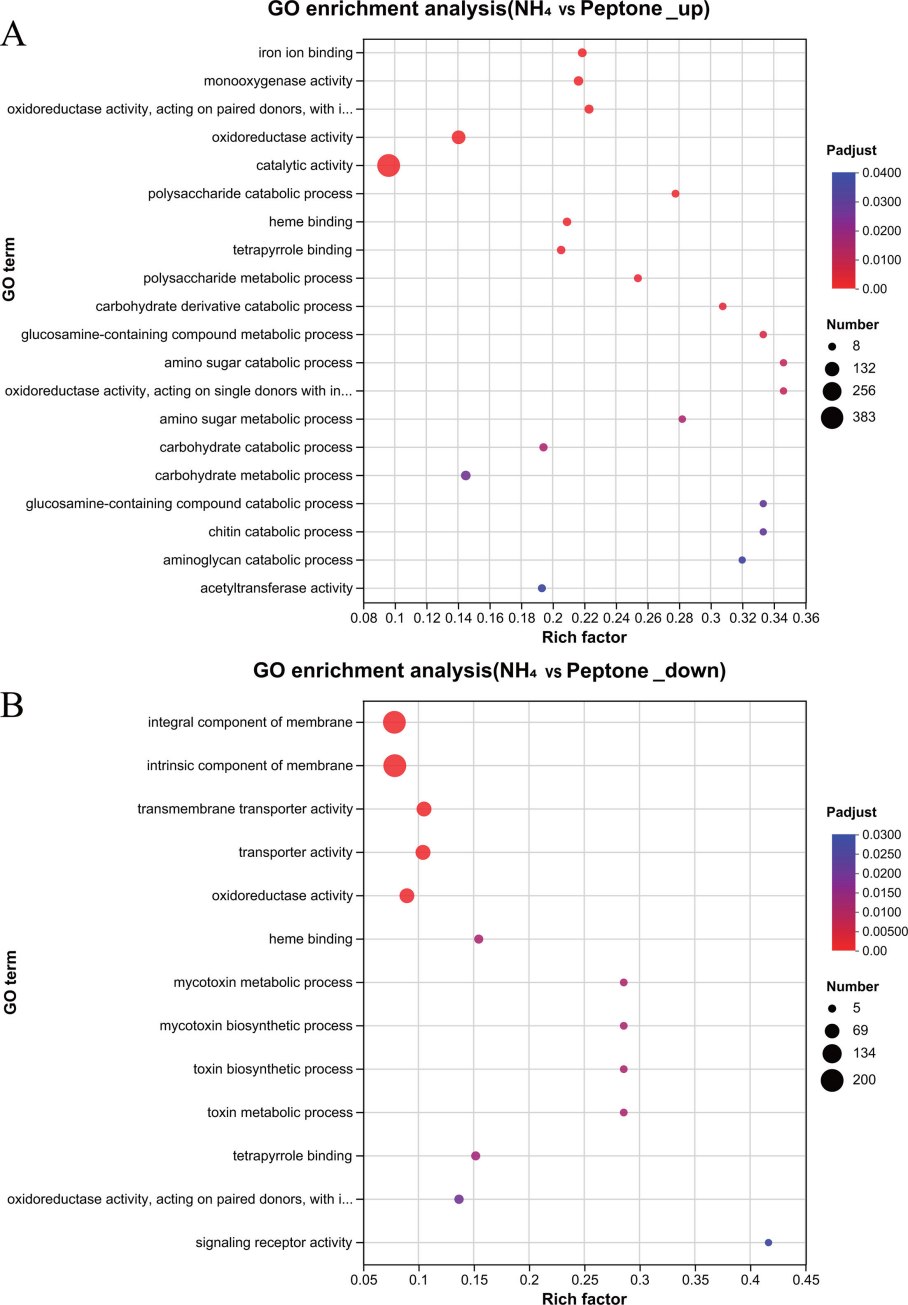
GO analysis of differentially expressed genes in NH_4_ vs peptone media. (**A**) Upregulated and (**B**) downregulated DEGs.

In the Gene Ontology (GO) analysis of these upregulated DEGs in the NH_4_ vs peptone medium, the following processes were significantly enriched: polysaccharide catabolic process, polysaccharide metabolic process, carbohydrate derivative catabolic process, carbohydrate metabolic process, glucosamine-containing compound metabolic process, and amino sugar catabolic process (Fig. 5A). This explains why *C. militaris* produced more polysaccharides in the NH_4_ vs peptone medium. The Clusters of Orthologous Groups (COG) analysis of the DEGs supported the conclusion of the GO analysis (Fig. S6). Similarly, in the GO analysis of upregulated DEGs in the NO_3_ vs peptone medium (Fig. S5B), the amino sugar catabolic process was significantly enriched, explaining why *C. militaris* produced more polysaccharides in the NO_3_ vs peptone medium. In the GO analysis of downregulated DEGs in the NH_4_ vs peptone medium, integral component of membrane, intrinsic component of membrane, and transmembrane transporter activity were significantly enriched (Fig. 5B), reflecting that the *C. militaris* mycelium biomass was lower in the NH_4_ vs peptone medium. In the Kyoto Encyclopedia of Genes and Genomes (KEGG) analysis of both the upregulated DEGs in the NH_4_ vs peptone medium (Fig. S7A) and the upregulated DEGs in the NO_3_ vs peptone medium (Fig. S7B), amino sugar and nucleotide sugar metabolism and nitrogen metabolism were significantly enriched. The increased cordycepin level in the NH_4_ or peptone medium, and the NO_3_ vs peptone medium (Fig. 4E), may be related to nitrogen metabolism. The upregulated CCM_09444 (NH_4_ or NO_3_ vs peptone), CCM_05697 (NH_4_ or NO_3_ vs peptone), CCM_02302 (NO_3_ vs peptone), and CCM_02951 (NO_3_ vs peptone) genes were all upregulated and were annotated as nitrogen metabolism genes.

As mentioned above, the cordycepin level was higher in the NH_4_ or NO_3_ vs peptone medium and the NH_4_ vs NO_3_ medium (Fig. 4E). We explored the potential cordycepin synthesis pathway based on the KEGG analysis results (Fig. S8). First, the CCM_02302 and CCM_02951 genes (which convert NO_3_ to NH_4_) were upregulated in the NO_3_ vs peptone medium. Second, CCM_09444 and CCM_05697 (which convert NH_4_ to glutamine) were upregulated in the NH_4_ or NO_3_ vs peptone medium. Third, CCM_06768 [which converts hypoxanthine nucleotide (IMP) to adenylosuccinate] and CCM_04436 (*cns1*), CCM_04437 (*cns2*), CCM_04438 (*cns3*), and CCM_00622 (which facilitate cordycepin synthesis) were upregulated in the NH_4_ or NO_3_ vs peptone medium and the NH_4_ vs NO_3_ medium (Fig. S8; Tables S3 and S5). The results suggest that culturing *C. militaris* in NH_4_ or NO_3_ medium increases the final cordycepin level by modulating the glutamine levels. Thus, the upregulation of various cordycepin synthesis genes in the NH_4_ or NO_3_ vs peptone medium and the NH_4_ vs NO_3_ medium promoted cordycepin synthesis, increasing the cordycepin level.

### Validation of gene expression patterns by RT-qPCR

We used RT-qPCR to verify the RNA-seq results regarding genes involved in cordycepin synthesis and nitrogen metabolism pathways in *C. militaris* cultured with different nitrogen sources. The RT-qPCR results and RNA-seq results were consistent (Tables S3 and S5).

### Co-culture of isolated bacterial strains and *C. militaris*

**Fig 6:**
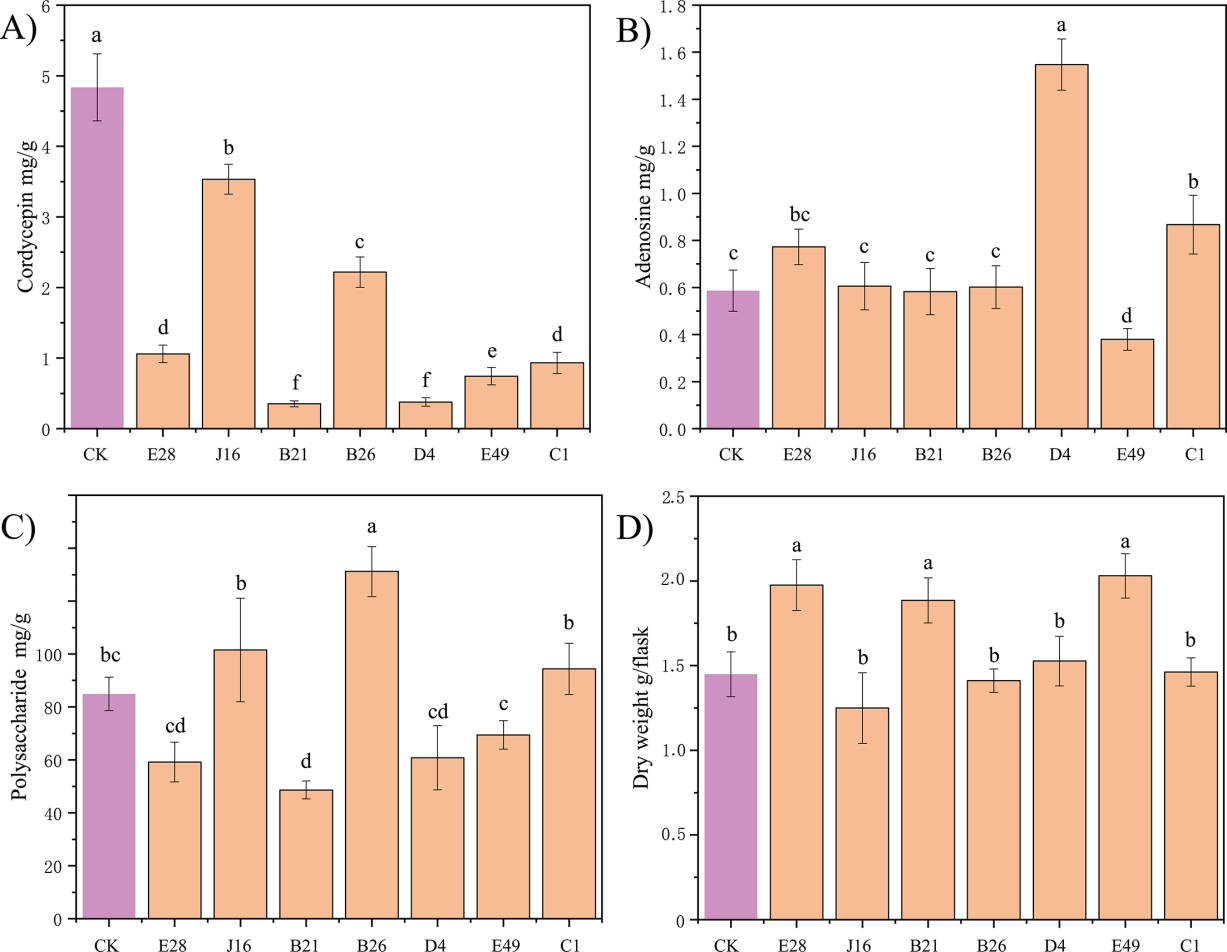
Effect of seven bacterial strains on secondary metabolites and biomass of *C. militaris*. (**A**) Cordycepin level, (**B**) adenosine level, (**C**) polysaccharide level, and (**D**) C. *militaris* mycelium biomass. Values are mean ± SD from three biological replicates. Different letters above the bars indicate significant differences (*P* < 0.05). E28, *Rhodococcus jostii*; J16, *Achromobacter marplatensi*s; B21, *Acinetobacter lwoffii*; B26, *Sphingobacterium multivorum*; D4, *Mycobacterium stephanolepidis*; E49, *Variovorax gossypii* E49; C1, *Pseudomonas protegens.*

None of the seven bacterial strains increased cordycepin synthesis by *C. militaris* (Fig. 6A). However, strain E28 (0.77 ± 0.08 mg/g) (*P* < 0.05) and strain D4 (1.55 ± 0.11 mg/g) significantly increased the adenosine production compared to the control (without co-culture with a bacterial strain) (0.59 ± 0.09 mg/g) (*P* < 0.01) (Fig. 6B), while strain B26 (131.20 ± 9.46 mg/g) significantly increased the extracellular polysaccharide level compared to the control (84.92 ± 6.29 mg/g) (*P <* 0.01) (Fig. 6C). Additionally, strain E28 (1.98 ± 0.15 g/flask), strain B21 (1.89 ± 0.13 g/flask), and strain E49 (2.03 ± 0.13 g/flask) significantly increased the C. *militaris* mycelium biomass compared to the control (1.45 ± 0.13 g/flask) (*P* < 0.05) (Fig. 6D). This indicates that the coexistence of microbes in the sclerotia influences the metabolism and growth of *C. militaris*.

Although none of the seven isolated bacterial strains increased the cordycepin level during co-culture with *C. militaris* (Fig. 6A), cordycepin increased when *C. militaris* was grown in the NH_4_ or NO_3_ vs peptone medium (Fig. 4E) due to bacterial conversion of the nitrogen compounds in the culture medium. In contrast, isolated bacterial strains E28, B21, and E49 increased the mycelium biomass (Fig. 6D), whereas bacterial conversion of nitrogen compounds in the culture medium did not increase the mycelium biomass (i.e., the mycelium biomass was significantly lower in the NH_4_ or NO_3_ vs peptone medium) (Fig. 4B). These results show that the isolated bacterial strains directly affected *C. militaris* growth via their own secretions, and bacteria can also indirectly affect *C. militaris* metabolites (such as cordycepin) through bacterial conversion of the nitrogen compounds in the culture medium. Bacterial strains J16, B26, and C1 increased the polysaccharide level when co-cultured with *C. militaris* (Fig. 6C), and bacterial conversion of the nitrogen compounds in the culture medium also increased the polysaccharide level of *C. militaris* (Fig. 4D). These results indicate that there are both direct and indirect ways to increase the polysaccharide level of *C. militaris*. In summary, when bacterial strains and *C. militaris* interact, both direct and indirect mechanisms (i.e., involving bacterial secretions or bacterial conversion of nitrogen compounds in the culture medium) can affect *C. militaris* growth and metabolite production.

## DISCUSSION

### Microbial community composition in *C. militaris* sclerotia is based on functional needs

Nitrogen is a key element for life, and microbes have evolved diverse strategies to obtain nitrogen from the environment. The most common strategy is to absorb NH_4_ and simple amino acids, which have a relatively low energy cost (20). Insect pupae contain abundant nitrogen sources, including organic nitrogen [mainly in the form of protein (21)] and inorganic nitrogen [including uric acid, urea, and ammonia (22)]. To avoid the toxic effects of ammonia produced by the catabolism of proteins and nucleic acids, living insects typically need to synthesize uric acid, which is then metabolized into urea to eliminate it from the body (23). Urea can be decomposed into NH_4_ (24), and NO_3_ can be converted to NH_4_ (25, 26). This results in higher NH_4_–N levels than NO_3_–N levels in insect remains. In a high-nitrogen environment such as in insect remains, microbes speed up the nitrogen cycle. In this way, a nutrient-rich habitat that is more conducive to *C. militaris* growth can be created. Therefore, *C. militaris* recruits and enriches relevant bacteria by releasing certain metabolites (27), a manifestation of bacteria–fungi interactions (28). The isolated bacterial strains played a role in nitrogen metabolism, based on their FAPROTAX-predicted functions, which were experimentally verified (Fig. 3; Table 1; Table S6). Additionally, the nitrogen metabolism capacity of these bacteria has been confirmed by other researchers (29–35).

A microbial community structure is not only the result of adaptation to the environment (36) but also reflects the functional requirements of the specific environment (37), and microbial community composition follows particular rules (38). For example, the initial nutritional status of the community (39), metabolite-based cross-feeding (40), and resource-competing microbial interactions all affect the composition of the community and its eventual functional characteristics (41). In addition, as the bacterial strains had multiple nitrogen metabolism functions and multiple strains performed the same functions (Table 1), the bacteria in the sclerotia clearly exhibited functional redundancy regarding nitrogen metabolism. The involvement of diverse microbes enhances the nitrogen conversion efficiency (42), improving environmental adaptability and system stability (43, 44). In summary, active nitrogen metabolism in living organisms is an evolutionary survival strategy (45), and *C. militaris* recruits and enriches microbes for specific functions.

### Bacteria in sclerotia can affect metabolite production in *C. militaris*

Cordycepin (C_10_H_13_N_5_O_3_) is an adenosine analog that can be converted from adenosine (C_10_H_13_N_5_O_4_) (46, 47). As cordycepin contains nitrogen, its synthesis requires a high nitrogen level. Within a certain range, the more abundant the nitrogen source, the greater the availability of basic raw materials for cordycepin synthesis.

Cordycepin synthesis was higher in the NH_4_ or NO_3_ vs peptone medium (Fig. 4E; Table S5), indicating that NH_4_ and NO_3_ increased the cordycepin synthesis efficiency.

Our results showed that enzymes, such as 5*'*-nucleotidase, adenylosuccinate synthetase, glutamine synthetase, glutamate dehydrogenase, and nitrate reductase, and the cns1, cns2, and *cns3* genes (involved near the end of cordycepin synthesis), were upregulated in the NH_4_ or NO_3_ vs peptone medium (Fig. S8; Table S5) (48). NO_3_–N can be converted to NH_4_–N by nitrate reductase (Fig. 4E; Table S5) (which can be produced by bacteria in the sclerotia), providing favorable conditions for cordycepin synthesis. NH_4_/ammonia can be converted to glutamine (a direct precursor of nucleosides such as adenosine) (19) by glutamine synthetase (49, 50) (which can also be produced by bacteria in the sclerotia), meaning that cordycepin synthesis can be regulated by the glutamine and glutamate pathways (Fig. S8) (51). A series of bacteria transformations in the *C. militaris* sclerotia can then be used to synthesize cordycepin from glutamine (Fig. S8). We inferred that when the NH_4_–N level is high (within a certain range), the cordycepin level is also increased. As to how wide this range is, further experiments are needed to clarify this.

Increased iron promotes cordycepin synthesis (52), which may be attributable to the impact of iron ions on enzymes. For example, iron-containing heme is an important cofactor involved in a variety of biological processes, including photosynthesis and respiration (53). It is also involved in oxygen transport and storage, electron transport, signal transduction, and micro-RNA processing (54). The GO analysis revealed that both iron ion binding and heme binding were enriched in upregulated DEGs in the NH_4_ vs NO_3_ medium (Fig. S5A) and the NH_4_ vs peptone medium (Fig. 5A). This finding further indicates that iron metabolism was regulated in the NH_4_ medium, which in turn facilitated cordycepin synthesis, so the cordycepin level was increased in the presence of NH_4_. Co-culture of the isolated bacterial strains with *C. militaris* did not increase the cordycepin level (Fig. 6). However, all the isolated bacterial strains demonstrated the ability to reduce NO_3_, and some had the ability to decompose urea into NH_4_ (Table 1). Furthermore, the cordycepin level was increased when *C. militaris* was grown in the NH_4_ or NO_3_ vs peptone medium due to bacterial conversion of nitrogen compounds in the culture medium (Fig. 4), that is, bacteria can increase the cordycepin level via an indirect pathway.

*Cordyceps* pigment has antioxidant effects (55) and can help fungi to tolerate sunlight and ultraviolet radiation (56, 57), so increased pigment indicates that *Cordyceps* has better environmental resistance and growth performance (58). *C. militaris* produced more carotenoid pigment and biomass (Fig. 4) in peptone vs NH_4_ or NO_3_ medium. Besides, in addition to NH_4_ and NO_3,_ there were other types of nitrogen in the sclerotia, such as organic nitrogen (Fig. 4A) (22). Based on only altering the nitrogen source, the results show that although adding NH_4_ to the medium promotes cordycepin synthesis, it had limitations regarding increasing the biomass and carotenoid pigment production. In other words, each nitrogen source had different effects, with different ratios between the various nitrogen sources (organic vs inorganic nitrogen, NH_4_ vs NO_3_, etc.) optimizing specific *C. militaris* growth and metabolism characteristics. If a specific product such as cordycepin or polysaccharide is required, NH_4_ should be added to the medium. This study mainly examined bacterial functions from the perspective of inorganic nitrogen conversion, and, in the future, the functions should be analyzed from the perspective of organic nitrogen conversion, which could involve studying protein decomposition into amino acids and promoting metabolite production.

### Conclusions

There are stable core microbes in the *C. militaris* sclerotia. These core microbes can create a more favorable growth environment for *C. militaris* directly (via secretions) and indirectly (by regulating the nitrogen metabolism in insect bodies), which can influence *C. militaris* growth and metabolite production.

## MATERIALS AND METHODS

### Sample collection and preparation

The *C. militaris* sclerotia samples and attached soil samples were collected in September every year from 2019 to 2023 in Tieling city (42°40*'*36*"*N–42°40*'*42*"*N, 124°40*'*24*"*E–124°40*'*28*"*E), Liaoning Province, China, at an altitude of 180–216 m. Each annual sample involved three insects in a mixed sample, that is, three biological replicates.

The *C. militaris* sclerotia samples were prepared using the method reported by Luo et al. (10). Briefly, the *C. militaris* sclerotia (the hardened insect pupa remains after infection with fungal mycelium) were rinsed with sterile water to remove the attached soil, soaked in 75% alcohol and 2% sodium hypochlorite three times for 20 s each time, and then rinsed with sterile water. The attached soil samples comprised the soil remaining adhering to the insect pupa remains when they were dug out of the soil, about 1 cm around the *C. militaris*. The sclerotia and attached soil samples were stored at −80°C prior to further processing and analysis.

### Total microbial DNA extraction and high-throughput DNA sequencing

The *C. militaris* samples were ground in liquid nitrogen, and then the total microbial DNA was extracted using an E.Z.N.A. Soil DNA Kit (Omega, USA) according to the manufacturer’s instructions. Next, the bacterial V3–V4 region was PCR amplified using the universal primers 338F (5*'* -ACTCCTACGGGAGGCAGCAGCAG-3′) and 806R (5′-GGACTACHVGGGTWTCTAAT-3′) (10), while the fungal V1–V2 region was PCR amplified using the universal primers ITS1 (5′-TCCGTAGGTGAACCTGCGG-3′) and ITS4 (5′-TCCTCCCCTTATTGATATGC-3′) (59). The reagents and conditions for PCR amplification using an ABI GeneAmp 9700 PCR instrument were consistent with our previous method (10). Briefly, the 20 µL PCR system contained 4 µL 5 × FastPfu Buffer, 2 µL dNTPs (2.5 mmol/L), 0.8 µL of each primer (5 µmol/L), 0.4 µL TransStart FastPfu DNA Polymerase, 0.2 µL bovine serum albumin (10 µg/µL), and 20 ng template DNA, supplemented with ddH_2_O to give a total volume of 20 µL. The PCR conditions were as follows: denaturation at 95°C for 30 s, annealing at 55°C for 30 s, and extension at 72°C for 45 s, followed by a final extension at 72°C for 10 min and holding at 10°C until the reaction was stopped. The amplified products were sequenced on an Illumina MiSeq sequencing platform by Shanghai Majorbio Biomedical Technology Co., Ltd.

Paired-end reads were spliced according to overlap relationships, and then quality control and filtering were conducted. Effective sequences were obtained by identifying samples according to the barcode and primer sequences at the ends of each sequence, and then optimized sequences were obtained by correcting the sequence direction. To obtain a representative operational taxonomic unit for each sequence, Uparse software (http://www.drive5.com/uparse/) was used based on 97% sequence similarity after chimera removal. Species annotation of the OTUs was performed using the Ribosomal Database Project database (https://sourceforge.net/projects/rdp-classifier/). The raw sequence reads were submitted to the National Center for Biotechnology Information (NCBI accession numbers: PRJNA722375, PRJNA849724, PRJNA965898, and PRJNA1077668).

### RNA extraction and RNA-seq

*C. militaris* strain CM20191001, which was obtained from the Institute of Fungal Resources of Guizhou University, was cultured at 25°C for 14 days on three different media with different nitrogen sources and then exposed to light for 3 days. Illumina NovaSeq 6000 sequencing was performed, with three biological replicates per nitrogen source treatment. The total RNA was extracted from the fresh *C. militaris* mycelia using a TRIzol Reagent Kit (Invitrogen, USA) according to the manufacturer’s instructions. The RNA concentration and purity were measured using a NanoDrop2000 system (Thermo Scientific, USA) and checked using RNase-free agarose gel electrophoresis. mRNA was isolated from the total RNA by A–T base pairing with the mRNA poly(A) tail using magnetic beads attached to oligo(dT) sequences. Single-strand cDNA was synthesized using the mRNA as a template, and then stable double-strand cDNA fragments were formed by double-strand synthesis. The purified double-strand cDNA fragments were end repaired, A tailed, and ligated to Illumina-sequencing adapters. The ligation reaction was purified using AMPure XP Beads (1.0×). The ligated cDNA fragments were then subjected to size selection by agarose gel electrophoresis and PCR amplified before sequencing. The clean reads were mapped to the *C. militaris* genome using TopHat v2.0.9 (60). The RNA-seq data were submitted to the Sequence Read Archive database (BioProject ID: PRJNA1047674).

Genes with a log2(fold change) ≥|1| and a false discovery rate <0.001 were identified as significant differentially expressed genes. The Kyoto Encyclopedia of Genes and Genomes, Gene Ontology, and COG databases were used to assign functions to the DEGs, using the Majorbio Cloud Platform (https://cloud.majorbio.com/page/tools/).

### RT-qPCR validation of RNA-seq results

The same RNA samples used for RNA-seq were used for RT-qPCR. The cDNA was synthesized using a reverse transcription kit (Vazyme Biotech, China). Briefly, a 20-µL reaction system was established with 50–2 µg of total RNA, which was then incubated at 50°C for 50 min and 85°C for 5 min to obtain the cDNA. Next, RT-qPCR was conducted using a TIB8600 fluorescence qPCR instrument (Triplex International Bioscience Co., Ltd.). The reference gene was 18S rRNA from C. *militaris*. The primers (Table S4) were synthesized by Shanghai Sangon Biotech. The RT-qPCR was conducted in a 20-µL PCR system comprising 2 µL cDNA (50 ng/µL), 10 µL Fast Start Essential DNA Green Master, 6 µL ddH_2_O, and 1 µL (10 µM) of each primer. The PCR conditions were as follows: predenaturation at 95°C for 5 min; 40 cycles of denaturation at 95°C for 15 s, 60°C for 30 s, and 72°C for 30 s; and a final extension at 72°C for 10 min. The relative gene expression was calculated using the 2^−ΔΔCt^ method.

### Isolation and identification of bacterial strains in *C. militaris* sclerotia

To isolate bacterial strains from *C. militaris* sclerotia, 1.0 g of *C. militaris* sclerotia sample was diluted with sterile water to 10^−2^, 10^−3^, and 10^−4^. Next, 100 µL was cultured at 28°C for 24 h in Luria–Bertani agar (10 g/L peptone, 5 g/L yeast extract, 10 g/L NaCl, and 15 g/L agar). Single bacterial colonies were selected, purified, and cultured three times, and then the 16S rRNA gene and *gyrB* genes were used for molecular identification (10). The primers for the 16S rRNA gene were 27F (5′-AGAGTTTGATCCTGGCTCAG-3′) and 1492R (5′-GGTTACCTTGTTACGACTT-3′), and the PCR amplification conditions were consistent with our previous method (10). The primers for *gyrB* were 5′-GAAGTCATCATGACCGTTCTGCAYGCNGGNGGNAARTTYGA-3′(F) and

5*'*-AGCAGGGTACGGATGTGTGCGAGCCRTCNACRTCNGCRTCNGTCAT-3*'*(R).

The PCR system contained 1 µL DNA template, 1 µL of each primer, 2.5 µL dNTPs, 2.5 µL PCR buffer, and 0.5 µL Taq polymerase, supplemented with ddH_2_O to give a total volume of 25 µL. The PCR conditions for 16S rRNA were as follows: predenaturation at 95°C for 3 min; 35 cycles of denaturation at 94°C for 30 s, annealing at 55°C for 30 s, and extension at 72°C for 30 s; and a final extension at 72°C for 10 min. The PCR conditions for *gyrB* were as follows: predenaturation at 94°C for 10 min; 35 cycles of denaturation at 94°C for 30 s, annealing at 52°C–58°C for 30 s, and extension at 72°C for 60 s; and a final extension at 72°C for 10 min. The amplified products were sequenced by Tsingke Biotechnology Co., Ltd. The bacterial sequence data were submitted to NCBI (accession numbers PP851015 to PP851021).

Next, the obtained 16S rRNA and *gyrB* gene sequences were analyzed using Basic Local Alignment Search Tool in NCBI. After determining each bacterial genus, different species belonging to the same genus were identified in the NCBI database to construct phylogenetic trees (with *Staphylococcus aureus* as the outgroup) using the neighbor-joining method in MEGA X software (10). To ensure as much consistency as possible regarding the species used for tree construction, the species selection for the *gyrB* gene was consistent with that for the 16 s rRNA gene. Additionally, the biochemical and physiological characteristics of the bacterial strains were identified using the bacterial biochemical identification strips HBIG05 and HBIG08 (Qingdao Hopebio Biotechnology Co., Ltd.) (Table S2). Their morphological and biochemical characteristics were assessed based on Bergey’s Manual of Systematic Bacteriology (Eighth Edition).

### *C. militaris* strain and media

*C. militaris* strain CM20191001, which was preserved at the Institute of Fungal Resources of Guizhou University, was used for assessing (i) *C. militaris* cultured in media with different nitrogen sources and (ii) co-culture of *C. militaris* with bacterial strains isolated from the *C. militaris* sclerotia.

“NH_4_,” “NO_3_,” and “peptone” media denote the three media with different nitrogen sources, which contained 3 g nitrogen in the form of ammonium sulfate [(NH_4_)_2_SO_4_], sodium nitrate (NaNO_3_), or peptone, respectively. The media also contained 30 g sucrose, 1 g K_2_HPO_4_, 0.5 g KCl, 0.2 g NaCl, 0.5 g MgSO_4_·7H_2_O, 0.01 g FeSO_4_, and 1 L H_2_O. For non-liquid medium, 20 g agar was added. Sabouraud liquid medium contained 40 g glucose, 10 g peptone, and 1 L H_2_O.

### Sporulation measurement

The *C. militaris* strain CM20191001 mycelia cultured on petri dishes containing media with different nitrogen sources for 28 days were scraped off, dried at 60°C, weighed, and then shaken with 20 mL 0.1% Tween to assess the sporulation. To reduce the statistical differences caused by biomass differences in different petri dishes, the spore yield was converted to conidia per gram.

### Determination of metabolite production and biomass

In the experiment involving media with different nitrogen sources, *C. militaris* strain CM20191001 was inoculated into 250 mL triangular shakers (100 mL liquid capacity) containing media with different nitrogen sources, cultured at 150 r/min at 25°C for 6 days, and then centrifuged at 8,000 r/min for 5 min. The supernatant was obtained to assess the polysaccharide, cordycepin, adenosine, and carotenoid levels. The mycelium pellets were dried at 60°C and weighed to calculate the biomass.

In the experiment involving co-culture of each of the seven bacterial strains with *C. militaris*, a previously described method (10) was used. Briefly, *C*. *militaris* strain CM20191001 was cultured in a triangular shaker containing Sabouraud liquid medium for 3 days, the bacterial suspension was added and cultured until day 7, and then the mixture was centrifuged at 8,000 r/min for 5 min. The supernatant was obtained to assess the polysaccharide, cordycepin, and adenosine levels. The mycelium biomass was calculated by drying the pellets. The cordycepin, adenosine, and polysaccharide levels in the supernatant were converted from grams per liter to milligrams per gram by dividing the quantity of cordycepin, adenosine, and polysaccharide by the *C. militaris* mycelium biomass.

The polysaccharide level was determined by the sulfuric acid–phenol method, according to the national agricultural standard NY/T 1676-2008, China. Briefly, polysaccharides were precipitated in ethanol, and the resulting furfural derivatives of the polysaccharides were dehydrated in concentrated sulfuric acid and condensed with phenol, forming orange/red compounds with a color intensity proportional to the concentration of polysaccharides in the solution. The cordycepin and adenosine levels were determined by high-performance liquid chromatography according to the national agricultural standard NY/T 2116-2012, China. An Agilent 1260 LC system and a C18 column were used with an acetonitrile:water (5:95, vol:vol) mobile phase at a flow rate of 1.0 mL/min, a column temperature of 35°C, a detection wavelength of 260 nm, and a sample volume of 10 µL. The carotenoid level was determined by using the acid-heat method to break the cell walls (61) and using acetone:petroleum ether (4:1, vol/vol) as an extraction agent. Briefly, *C. militaris* mycelia were cultured on plates containing different nitrogen source media for 21 days to obtain 0.3 g sample. Next, 4.5 mL 1 mol/L HCl was added to each 0.3 g sample, followed by adding 0.1% butylated hydroxytoluene to prevent oxidation, soaking for 30 min, boiling for 4 min, and immediately cooling on ice and centrifuging at 5,000 r/min for 10 min. The supernatant was discarded, and the pellet was washed twice followed by centrifuging at 8,000 r/min (avoiding light). The supernatant was discarded, 4.5 mL acetone:petroleum ether (4:1, vol/vol) was added, the mixture was subjected to extraction twice for 30 min each time, and the extraction solutions were combined. The absorbance at 445 nm was then determined using an ultraviolet spectrophotometer, and the carotenoid level was calculated using the following formula:


$$Carotenoid\;production\;(mg/g)=\frac{A\times V\times D\times 1,000}{0.16\times W},$$

where *A* is the absorbance value; *V* is the volume of the extraction reagent; *D* is the dilution ratio; 1,000 is the substitution coefficient for converting micrograms to milligrams; 0.16 is the extinction coefficient; and *W* is the *C. militaris* sample dry weight (g).

### Determination of NO_3_ reduction ability, urea decomposition ability, and total nitrogen, NO_3_–N, and NH_4_–N

NO_3_ reduction ability of the isolated bacterial strains was assessed using an NO_3_ reduction kit (HB8282, Haibo Bio). Urea decomposition ability of the isolated bacterial strains was assessed using the phenol red indicator method (62). Total nitrogen, NO_3_–N, and NH_4_–N in the *C. militaris* sclerotia were determined according to LY/T 1228–2015, China.

### Data analysis

The VennDiagram package in R v3.6.3 was used to construct Venn diagrams (63), and the core microbes in the sclerotia were assessed using the Tutools platform (http://cloudtutu.com.cn/), a free online data analysis website. Functional prediction regarding the bacteria in the *C. militaris* sclerotia and attached soil samples was conducted using FAPROTAX (64). Histograms and boxplots were visualized using OriginPro2018.

A co-occurrence network of microbial OTUs was constructed based on OTUs with a relative abundance >0.05%. Spearman’s correlation coefficients (*R*) between the OTUs were calculated using the psych R package, and OTUs with *R* >0.75 and *P* < 0.05 were retained for the co-occurrence network to reveal the interactions among the OTUs. Finally, Gephi v0.9.2 was used to visualize the co-occurrence network (65).

Alpha diversity indexes (Shannon’s diversity index, Simpson’s diversity index, Chao1 richness estimator, and ACE richness estimator) were calculated using the Majorbio Cloud Platform (https://cloud.majorbio.com/page/tools/).

Values are expressed as mean ± standard deviation of three replicates. Analysis of variance followed by Duncan’s multiple range test was used to determine significant differences between means. *P* < 0.05 was considered to indicate a significant difference.

## Data Availability

The high-throughput data are numbered PRJNA722375, PRJNA849724, PRJNA965898, and PRJNA1077668 in the NCBI. The accession numbers of bacterial sequences in NCBI are PP851015 to PP851021. The transcriptome data were deposited in the NCBI Sequence Read Archive (SRA) under bioproject PRJNA1047674.

## SUPPLEMENTAL MATERIAL

Supplemental figures and tables spectrum.01053-24-s0001.pdf Fig. S1 to S8; Tables S1 to S6.

ASM does not own the copyrights to Supplemental Material that may be linked to, or accessed through, an article. The authors have granted ASM a non-exclusive, world-wide license to publish the Supplemental Material files. Please contact the corresponding author directly for reuse.
