# Supplementary material for: Core microbes in Cordyceps militaris sclerotia and their nitrogen metabolism-related ecological functions
Source: Microbiol Spectr. 2024 Aug 20;12(10):e01053-24. doi: 10.1128/spectrum.01053-24 (PMC11448085; doi:10.1128/spectrum.01053-24)
Supplement: Supplemental figures and tables — Fig. S1 to S8; Tables S1 to S6. [file spectrum.01053-24-s0001.pdf]

Core microbes in *Cordyceps militaris* sclerotia and their nitrogen metabolism-related ecological functions

Supplemental material

Fig. S1 Co-occurrence network of bacterial OTUs co-existing with *C.militaris*.

Fig. S2 Phylogenetic tree of bacterial strains isolated from *C.militaris* sclerotia.

Fig. S3. Effects of nitrogen sources on *C. militari*s carotenoid production.

Fig. S4 Volcano plots.

Fig. S5 GO analysis of upregulated differentially expressed genes (DEGs).

Fig. S6 Distributions and numbers of differentially expressed genes (DEGs) in COG functional categories.

Fig. S7 The KEGG analysis of differentially expressed genes (DEGs).

Fig. S8 Possible cordycepin synthesis pathway.

Table S1 Classification and abundance of coexist 67 OTUs coexisting with *C. militaris* in sclerotia.

Table S2 Biochemical analysis and morphological characteristics of bacteria isolated from *C. militaris* sclerotia.

Table S3 Transcriptomic regulation of genes.

Table S4 Primers used for RT-qPCR.

Table S5 RT-qPCR validation results.

Table S6 Abundance and predicted functions of 47 genera in sclerotia and in attached soil and their predicted function.

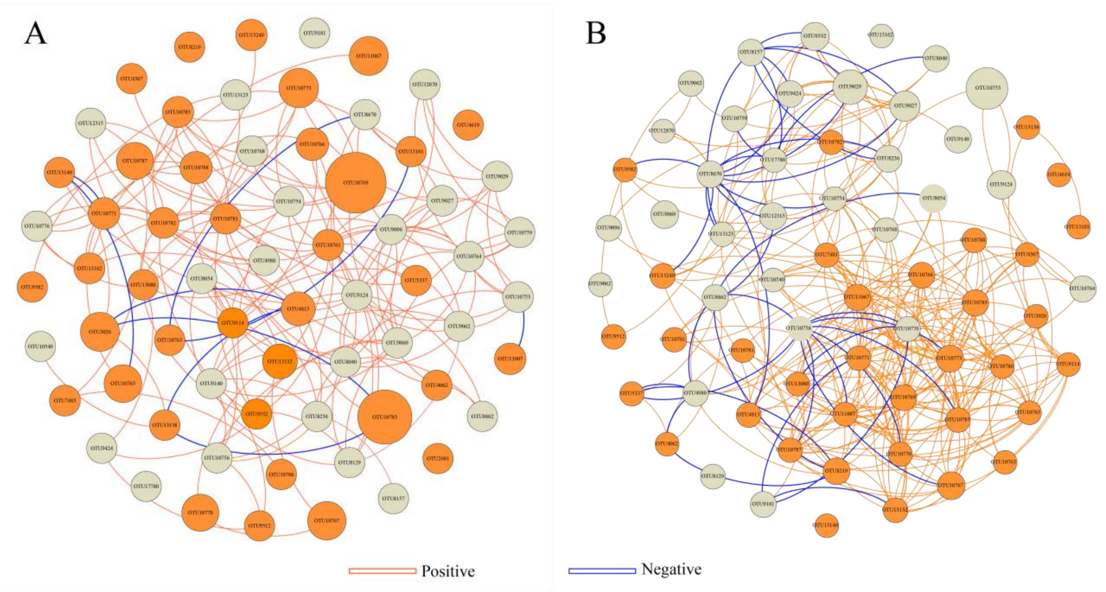

Fig. S1. Co-occurrence network of bacterial OTUs co-existing with *C.militaris*. (A) Bacterial 67 OTUs in *C. militaris* sclerotia; (B) Bacterial OTUs in attached soil. The orange dot indicates that the relative abundance of genus in the sclerotia is higher than that in the corresponding attached soil, and the yellow dot is lower. OTU classification information is in Table S1.

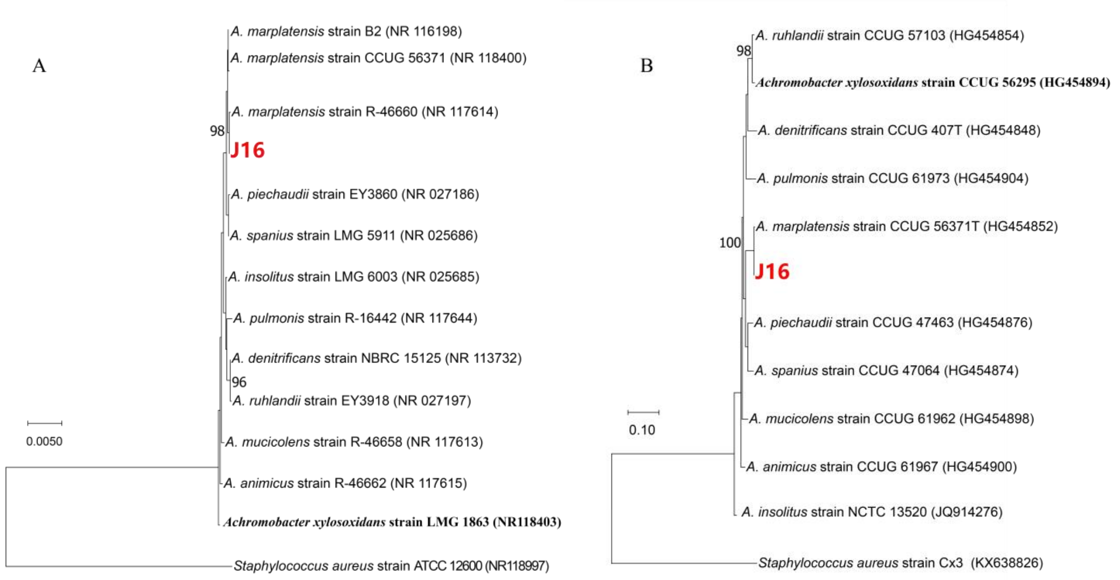
A
A. marplatensis strain B2 (NR 116198)
A. marplatensis strain CCUG 56371 (NR 118400)
A. marplatensis strain R-46660 (NR 117614)
98
J16
A. piechaudii strain EY3860 (NR 027186)
A. spanius strain LMG 5911 (NR 025686)
A. insolitus strain LMG 6003 (NR 025685)
A. pulmonis strain R-16442 (NR 117644)
A. denitrificans strain NBRC 15125 (NR 113732)
96
A. ruhlandii strain EY3918 (NR 027197)
0.0050
A. mucicolens strain R-46658 (NR 117613)
A. animicus strain R-46662 (NR 117615)
Achromobacter xylosoxidans strain LMG 1863 (NR118403)
Staphylococcus aureus strain ATCC 12600 (NR118997)
B
98
A. ruhlandii strain CCUG 57103 (HG454854)
Achromobacter xylosoxidans strain CCUG 56295 (HG454894)
A. denitrificans strain CCUG 407T (HG454848)
A. pulmonis strain CCUG 61973 (HG454904)
100
A. marplatensis strain CCUG 56371T (HG454852)
J16
A. piechaudii strain CCUG 47463 (HG454876)
A. spanius strain CCUG 47064 (HG454874)
0.10
A. mucicolens strain CCUG 61962 (HG454898)
A. animicus strain CCUG 61967 (HG454900)
A. insolitus strain NCTC 13520 (JQ914276)
Staphylococcus aureus strain Cx3 (KX638826)

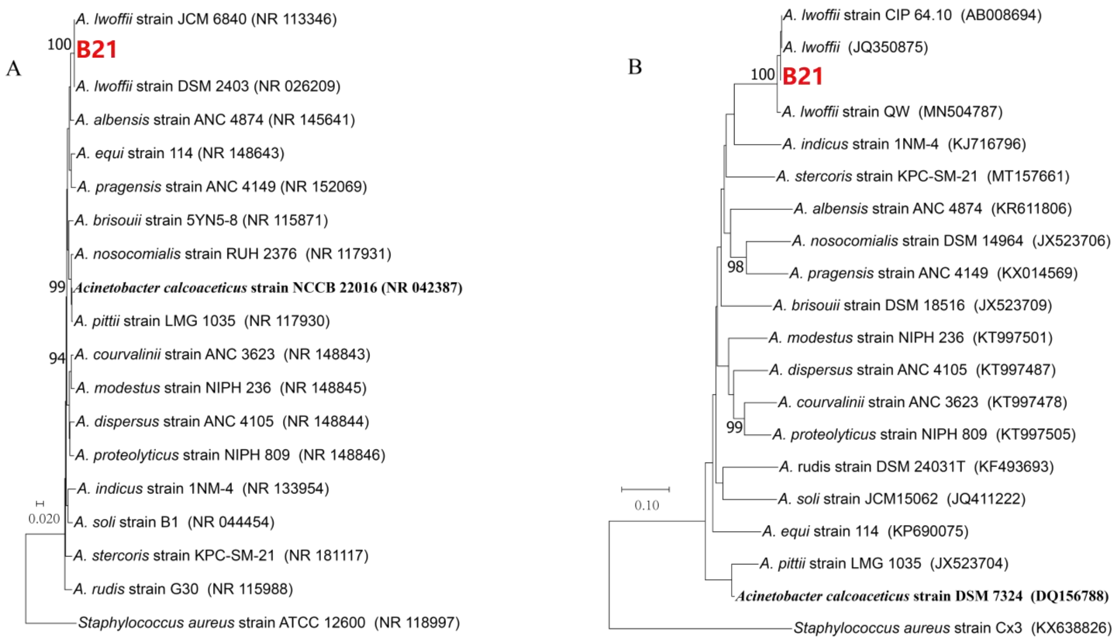
A
A. lwoffii strain JCM 6840 (NR 113346)
100
B21
A. lwoffii strain DSM 2403 (NR 026209)
A. albensis strain ANC 4874 (NR 145641)
A. equi strain 114 (NR 148643)
A. pragensis strain ANC 4149 (NR 152069)
A. brisouii strain 5YN5-8 (NR 115871)
A. nosocomialis strain RUH 2376 (NR 117931)
99
Acinetobacter calcoaceticus strain NCCB 22016 (NR 042387)
A. pittii strain LMG 1035 (NR 117930)
94
A. courvalinii strain ANC 3623 (NR 148843)
A. modestus strain NIPH 236 (NR 148845)
A. dispersus strain ANC 4105 (NR 148844)
A. proteolyticus strain NIPH 809 (NR 148846)
A. indicus strain 1NM-4 (NR 133954)
0.020
A. soli strain B1 (NR 044454)
A. stercoris strain KPC-SM-21 (NR 181117)
A. rudis strain G30 (NR 115988)
Staphylococcus aureus strain ATCC 12600 (NR 118997)
B
A. lwoffii strain CIP 64.10 (AB008694)
A. lwoffii (JQ350875)
100
B21
A. lwoffii strain QW (MN504787)
A. indicus strain 1NM-4 (KJ716796)
A. stercoris strain KPC-SM-21 (MT157661)
A. albensis strain ANC 4874 (KR611806)
A. nosocomialis strain DSM 14964 (JX523706)
98
A. pragensis strain ANC 4149 (KX014569)
A. brisouii strain DSM 18516 (JX523709)
A. modestus strain NIPH 236 (KT997501)
A. dispersus strain ANC 4105 (KT997487)
A. courvalinii strain ANC 3623 (KT997478)
99
A. proteolyticus strain NIPH 809 (KT997505)
A. rudis strain DSM 24031T (KF493693)
0.10
A. soli strain JCM15062 (JQ411222)
A. equi strain 114 (KP690075)
A. pittii strain LMG 1035 (JX523704)
Acinetobacter calcoaceticus strain DSM 7324 (DQ156788)
Staphylococcus aureus strain Cx3 (KX638826)

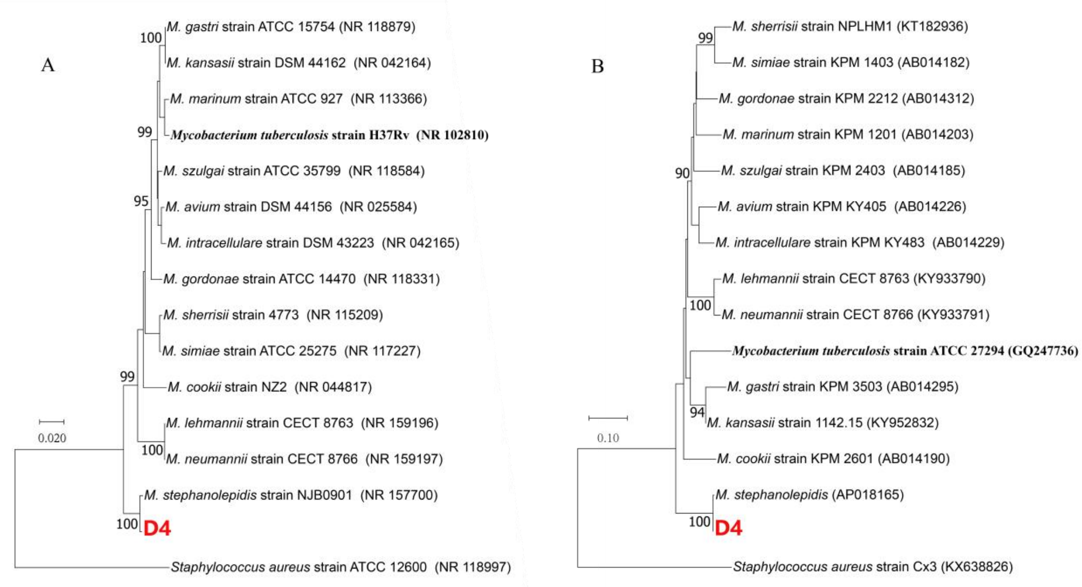
A
M. gastri strain ATCC 15754 (NR 118879)
M. kansasii strain DSM 44162 (NR 042164)
M. marinum strain ATCC 927 (NR 113366)
Mycobacterium tuberculosis strain H37Rv (NR 102810)
M. szulgai strain ATCC 35799 (NR 118584)
M. avium strain DSM 44156 (NR 025584)
M. intracellulare strain DSM 43223 (NR 042165)
M. gordonae strain ATCC 14470 (NR 118331)
M. sherrisii strain 4773 (NR 115209)
M. simiae strain ATCC 25275 (NR 117227)
M. cookii strain NZ2 (NR 044817)
M. lehmannii strain CECT 8763 (NR 159196)
M. neumannii strain CECT 8766 (NR 159197)
M. stephanolepidis strain NJB0901 (NR 157700)
D4
Staphylococcus aureus strain ATCC 12600 (NR 118997)
0.020
100
99
95
99
100
100
B
M. sherrisii strain NPLHM1 (KT182936)
M. simiae strain KPM 1403 (AB014182)
M. gordonae strain KPM 2212 (AB014312)
M. marinum strain KPM 1201 (AB014203)
M. szulgai strain KPM 2403 (AB014185)
M. avium strain KPM KY405 (AB014226)
M. intracellulare strain KPM KY483 (AB014229)
M. lehmannii strain CECT 8763 (KY933790)
M. neumannii strain CECT 8766 (KY933791)
Mycobacterium tuberculosis strain ATCC 27294 (GQ247736)
M. gastri strain KPM 3503 (AB014295)
M. kansasii strain 1142.15 (KY952832)
M. cookii strain KPM 2601 (AB014190)
M. stephanolepidis (AP018165)
D4
Staphylococcus aureus strain Cx3 (KX638826)
0.10
99
90
100
94
100

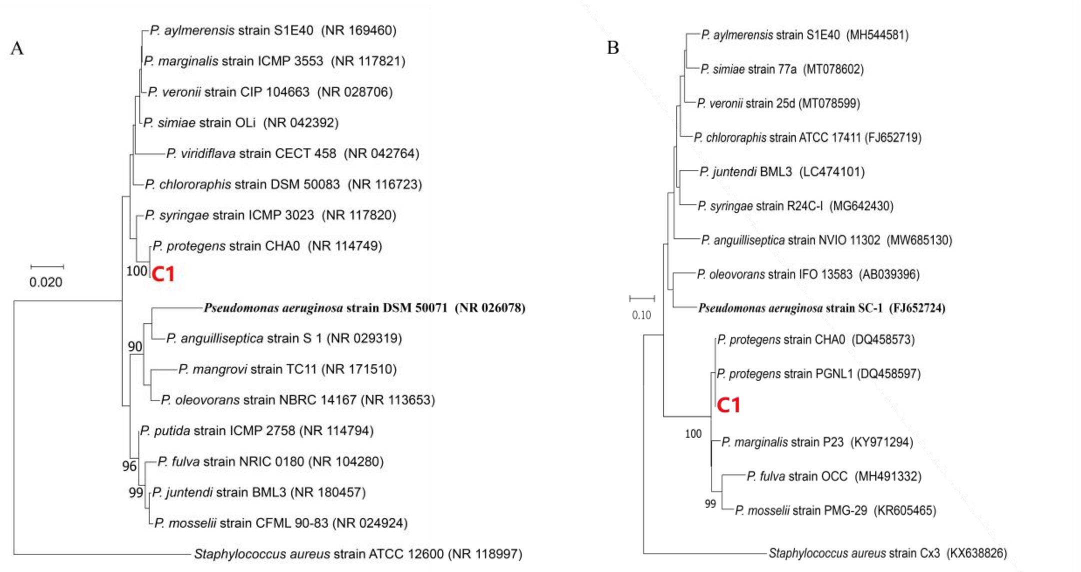
A
P. aylmerensis strain S1E40 (NR 169460)
P. marginalis strain ICMP 3553 (NR 117821)
P. veronii strain CIP 104663 (NR 028706)
P. simiae strain OLi (NR 042392)
P. viridiflava strain CECT 458 (NR 042764)
P. chlororaphis strain DSM 50083 (NR 116723)
P. syringae strain ICMP 3023 (NR 117820)
P. protegens strain CHA0 (NR 114749)
C1
Pseudomonas aeruginosa strain DSM 50071 (NR 026078)
P. anguilliseptica strain S 1 (NR 029319)
P. mangrovi strain TC11 (NR 171510)
P. oleovorans strain NBRC 14167 (NR 113653)
P. putida strain ICMP 2758 (NR 114794)
P. fulva strain NRIC 0180 (NR 104280)
P. juntendi strain BML3 (NR 180457)
P. mosselii strain CFML 90-83 (NR 024924)
Staphylococcus aureus strain ATCC 12600 (NR 118997)
0.020
100
90
96
99
B
P. aylmerensis strain S1E40 (MH544581)
P. simiae strain 77a (MT078602)
P. veronii strain 25d (MT078599)
P. chlororaphis strain ATCC 17411 (FJ652719)
P. juntendi BML3 (LC474101)
P. syringae strain R24C-I (MG642430)
P. anguilliseptica strain NVIO 11302 (MW685130)
P. oleovorans strain IFO 13583 (AB039396)
Pseudomonas aeruginosa strain SC-1 (FJ652724)
P. protegens strain CHA0 (DQ458573)
P. protegens strain PGNL1 (DQ458597)
C1
P. marginalis strain P23 (KY971294)
P. fulva strain OCC (MH491332)
P. mosselii strain PMG-29 (KR605465)
Staphylococcus aureus strain Cx3 (KX638826)
0.10
100
99

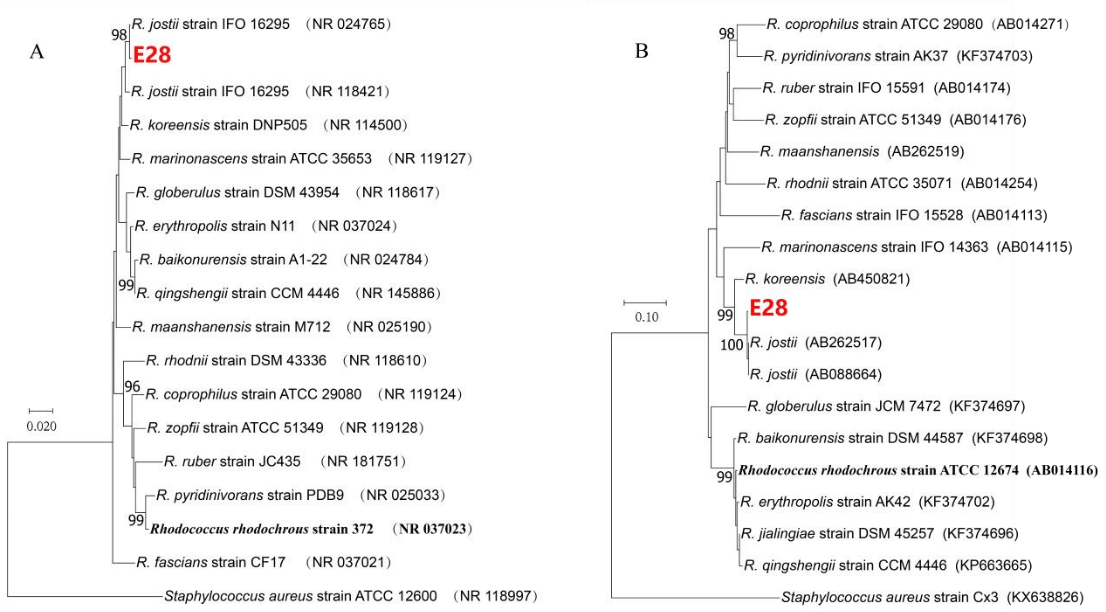
A
R. jostii strain IFO 16295 (NR 024765)
E28
R. jostii strain IFO 16295 (NR 118421)
R. koreensis strain DNP505 (NR 114500)
R. marinonascens strain ATCC 35653 (NR 119127)
R. globerulus strain DSM 43954 (NR 118617)
R. erythropolis strain N11 (NR 037024)
R. baikonurensis strain A1-22 (NR 024784)
R. qingshengii strain CCM 4446 (NR 145886)
R. maanshanensis strain M712 (NR 025190)
R. rhodnii strain DSM 43336 (NR 118610)
R. coprophilus strain ATCC 29080 (NR 119124)
R. zopfii strain ATCC 51349 (NR 119128)
R. ruber strain JC435 (NR 181751)
R. pyridinivorans strain PDB9 (NR 025033)
Rhodococcus rhodochrous strain 372 (NR 037023)
R. fascians strain CF17 (NR 037021)
Staphylococcus aureus strain ATCC 12600 (NR 118997)
0.020
98
99
96
99
B
R. coprophilus strain ATCC 29080 (AB014271)
R. pyridinivorans strain AK37 (KF374703)
R. ruber strain IFO 15591 (AB014174)
R. zopfii strain ATCC 51349 (AB014176)
R. maanshanensis (AB262519)
R. rhodnii strain ATCC 35071 (AB014254)
R. fascians strain IFO 15528 (AB014113)
R. marinonascens strain IFO 14363 (AB014115)
R. koreensis (AB450821)
E28
R. jostii (AB262517)
R. jostii (AB088664)
R. globerulus strain JCM 7472 (KF374697)
R. baikonurensis strain DSM 44587 (KF374698)
Rhodococcus rhodochrous strain ATCC 12674 (AB014116)
R. erythropolis strain AK42 (KF374702)
R. jialingiae strain DSM 45257 (KF374696)
R. qingshengii strain CCM 4446 (KP663665)
Staphylococcus aureus strain Cx3 (KX638826)
0.10
98
99
100
99

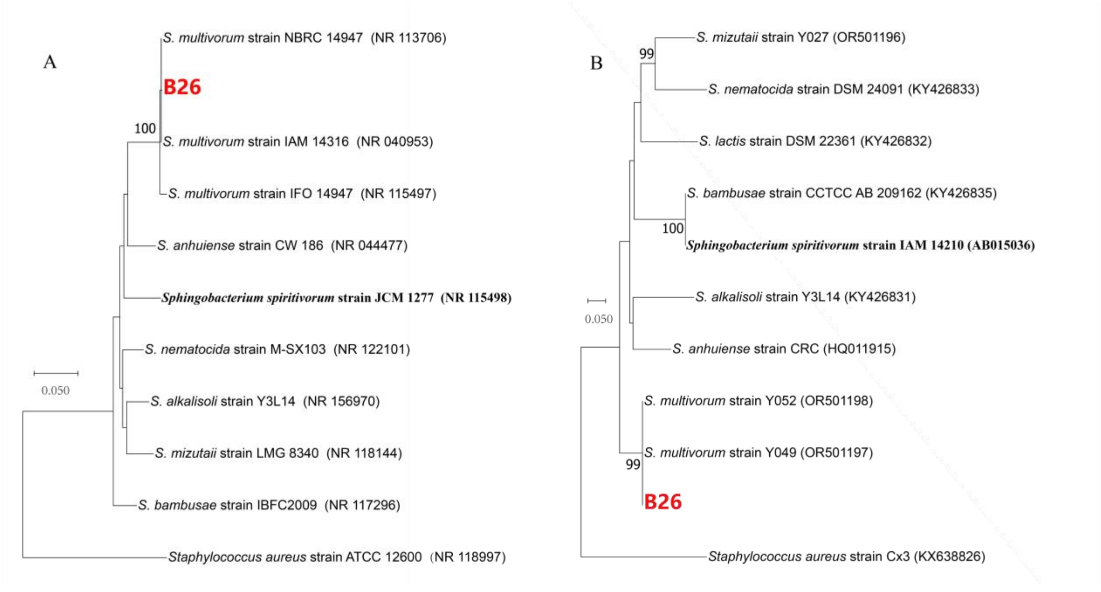

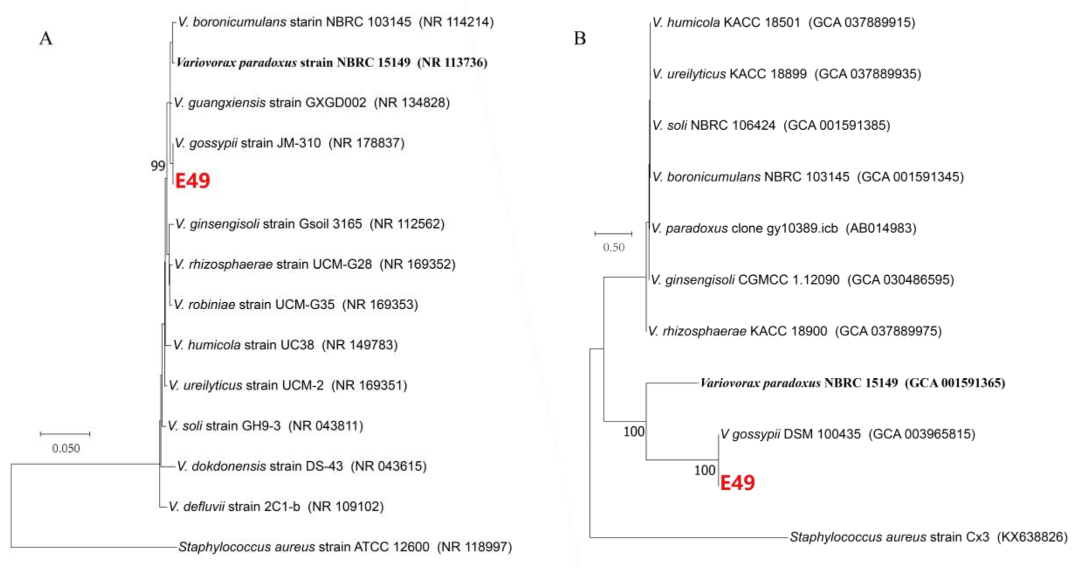

Fig. S2. Phylogenetic trees of bacterial strains isolated from *C.militaris* sclerotia. (A) 16S rRNA (B) *gyrB* gene. The bold font is the type strain, such as: *Achromobacter xylosoxidans*, *Acinetobacter calcoaceticus*, *Mycobacterium tuberculosis*, *Pseudomonas aeruginosa*, *Rhodococcus rhodochrous*, *Sphingobacterium spiritivorum*, *Variovorax paradoxus*. In parentheses is the GenBank accession number of the gene sequence.

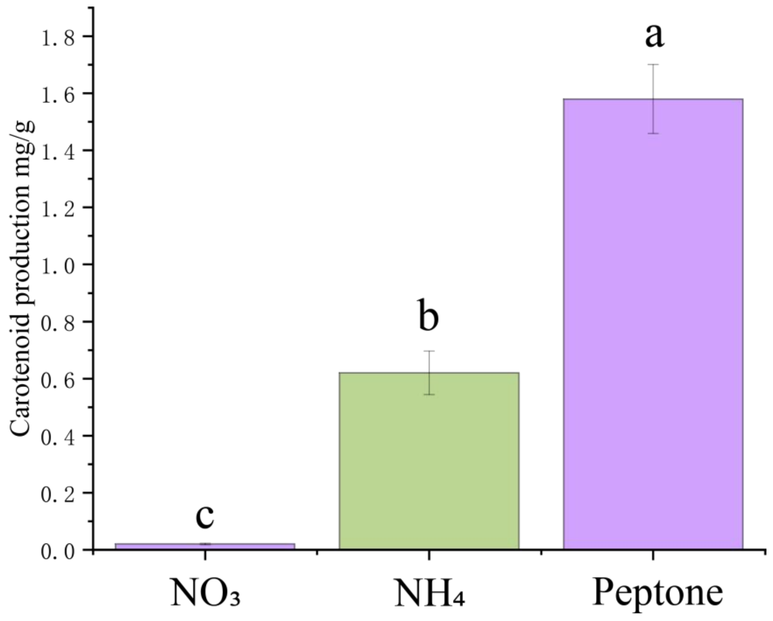

Fig. S3. Effects of nitrogen sources on *C. militaris* carotenoid production. Different letters above the bars indicate significant differences ($P < 0.05$).

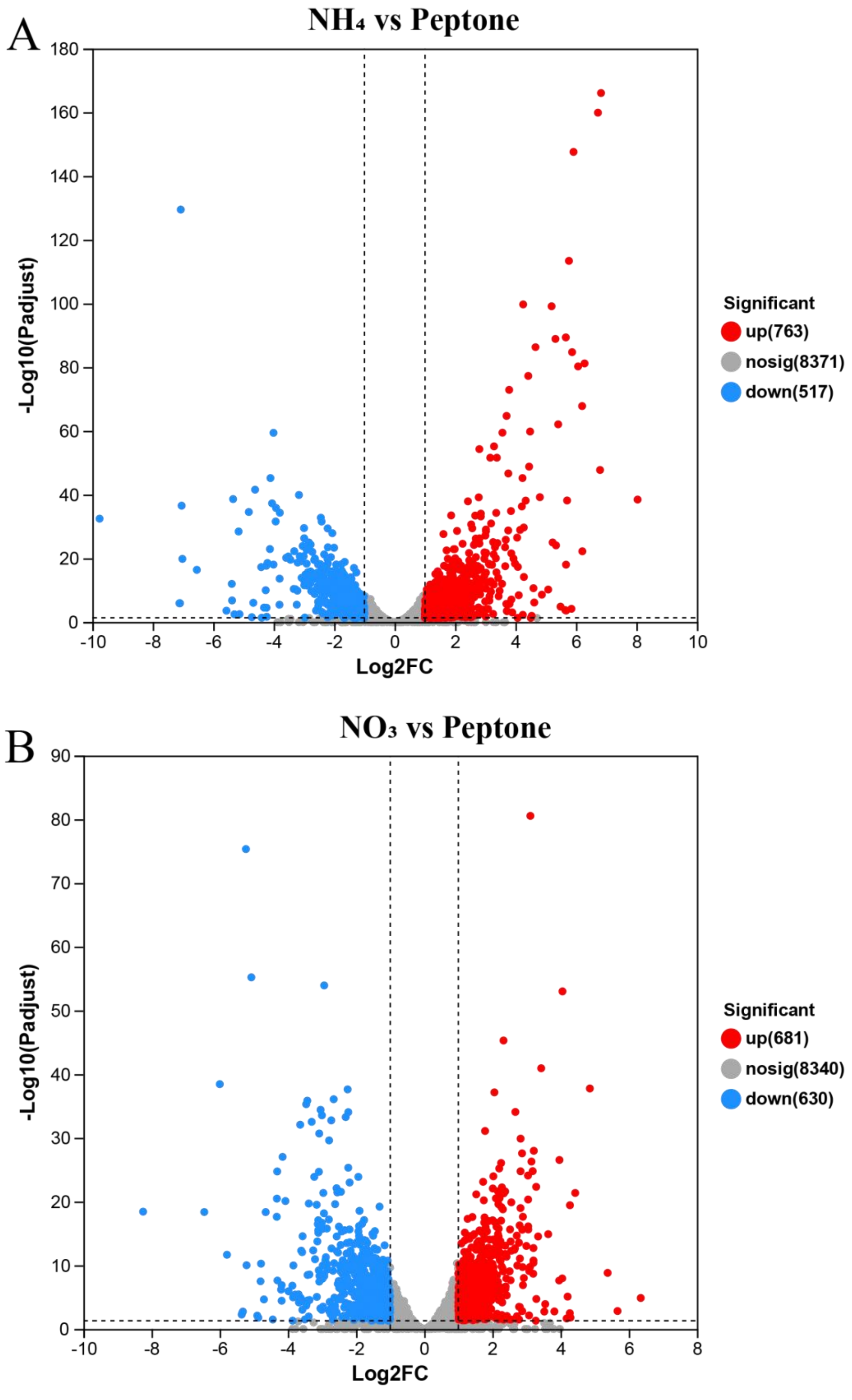

Fig. S4. Volcano plots. (A) NH_4 vs peptone; (B) NO_3 vs peptone. Red dots represent upregulated genes and blue dots represent downregulated genes.

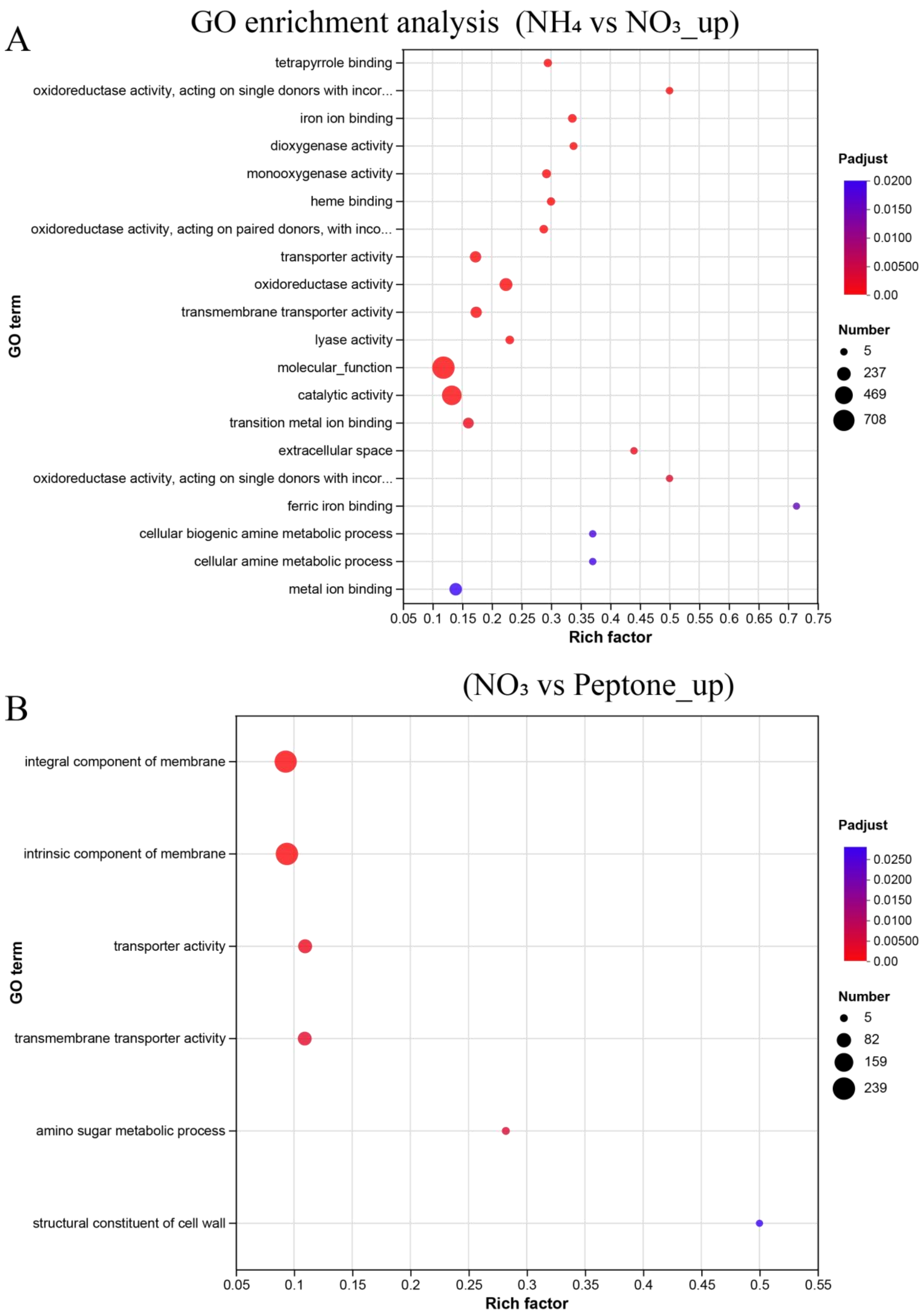

Fig. S5. GO analysis of upregulated differentially expressed genes (DEGs). (A) NH_4 vs NO_3; (B) NO_3 vs peptone.

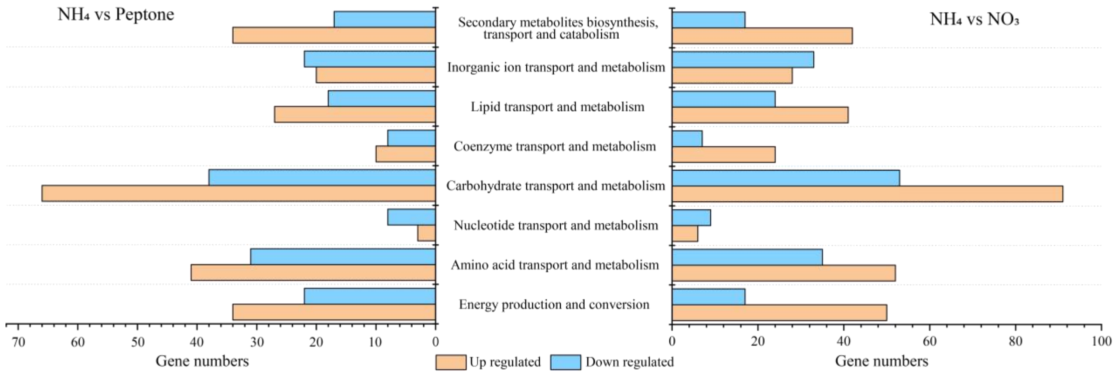

Fig. S6. Distributions and numbers of differentially expressed genes (DEGs) in COG functional categories.

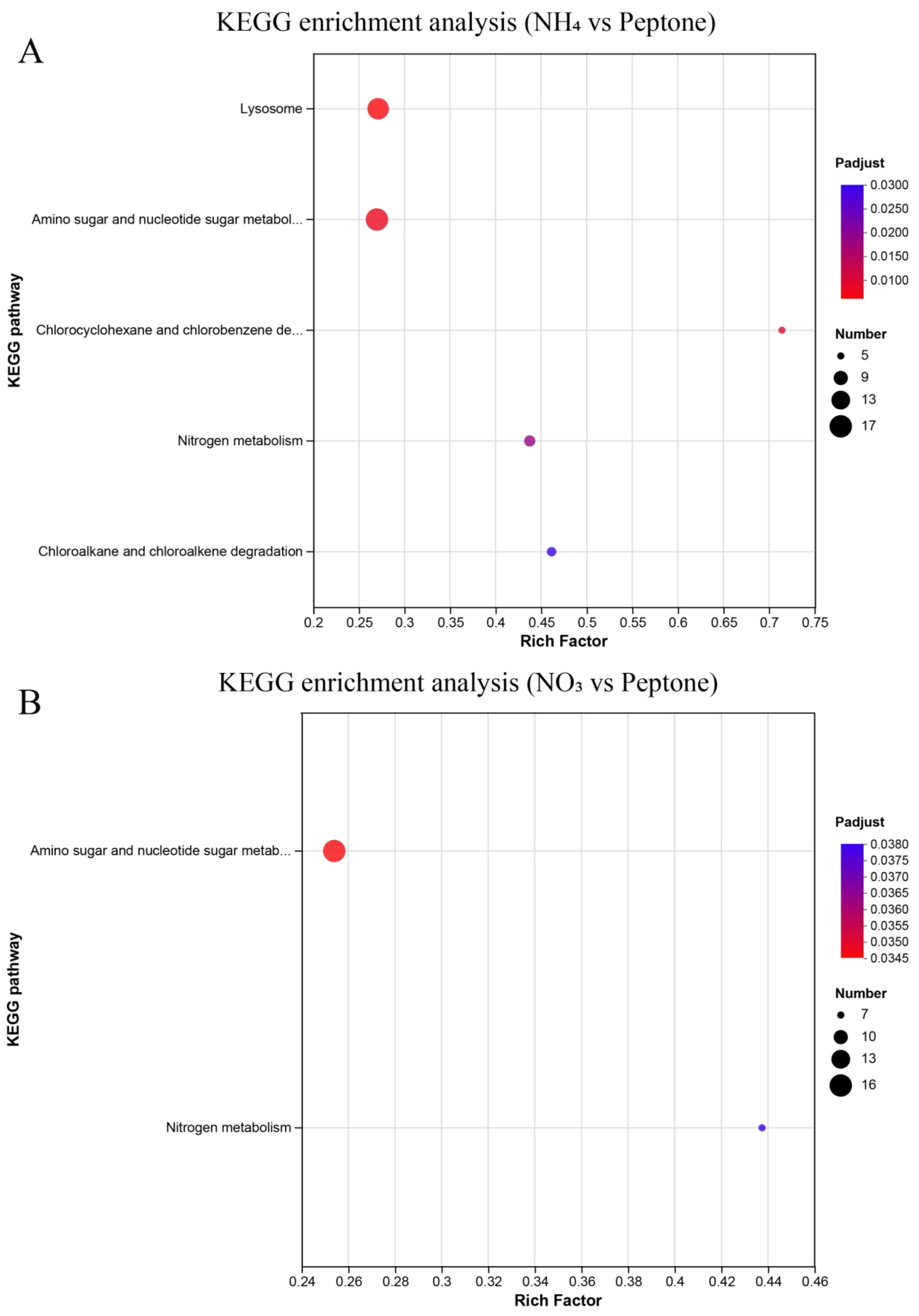

Fig. S7. The KEGG analysis of differentially expressed genes (DEGs).

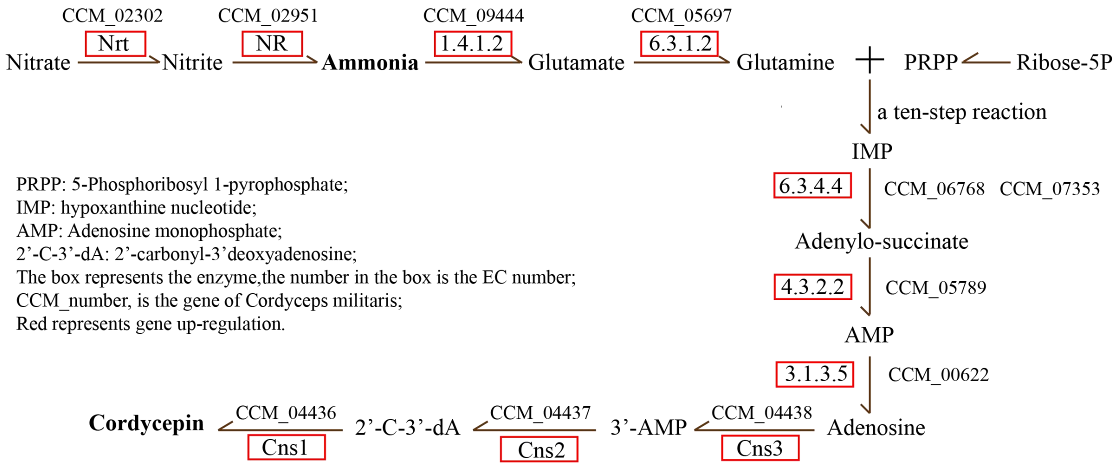

Fig. S8. Possible cordycepin synthesis pathway. Each box represents an enzyme. Each number in a box is an Enzyme Commission (EC) number. CCM_number indicates a *C. militaris* gene. Red represents upregulation.

Table S1 Classification and abundance of coexist 67 OTUs coexisting with *C. militaris* in sclerotia.

OTU ID	Taxonomy	Relative abundance	
		CMS	AS
OTU9181	p__Actinobacteriota;c__Acidimicrobiia;o__IMCC26256;f__unclassified_o__IMCC26256;g__unclassified_o__IMCC26256;	0.00019	0.00364
OTU10771	p__Proteobacteria;c__Gammaproteobacteria;o__Xanthomonadales;f__Xanthomonadaceae;g__Stenotrophomonas;	0.02478	0.00138
OTU10770	p__Proteobacteria;c__Gammaproteobacteria;o__Burkholderiales;f__Alcaligenaceae;g__Achromobacter;	0.11832	0.00079
OTU10773	p__Proteobacteria;c__Alphaproteobacteria;o__Rhizobiales;f__Rhizobiaceae;g__Ensifer;	0.16121	0.00535
OTU11007	p__Proteobacteria;c__Alphaproteobacteria;o__Rhizobiales;f__Rhizobiaceae;g__Ochrobactrum;	0.02037	0.00019

OTU8219	p__Actinobacteriota;c__Acidimicrobiia;o__Microtrichales;f__Ilumatobacteraceae;g__unclassified_f__Ilumatobacteraceae;	0.00019	0.00496
OTU10776	p__Bacteroidota;c__Bacteroidia;o__Flavobacteriales;f__Weeksellaceae;g__Chryseobacterium;	0.00528	0.00196
OTU10786	p__Proteobacteria;c__Alphaproteobacteria;o__Azospirillales;f__Inquilinaceae;g__Inquilinus;	0.00343	0.00019
OTU10787	p__Proteobacteria;c__Alphaproteobacteria;o__Rhizobiales;f__Labraceae;g__Labrys;	0.11786	0.00110
OTU10781	p__Proteobacteria;c__Alphaproteobacteria;o__Rhizobiales;f__Xanthobacteraceae;g__Rhodopseudomonas;	0.00217	0.00015
OTU10782	p__Proteobacteria;c__Alphaproteobacteria;o__Rhizobiales;f__Beijerinckiaceae;g__Bosea;	0.00915	0.00096
OTU107	p__Actinobacteriota;c__Actinobacteria;o__Corynebacteriales;f__Nocardiaceae;g__Rhodococcus;s__Rhod	0.41957	0.00434

83	ococcus_erythropolis		
OTU10788	p__Proteobacteria;c__Gammaproteobacteria;o__Pseudomonadales;f__Pseudomonadaceae;g__Pseudomonas;	0.03259	0.00149
OTU4619	p__Proteobacteria;c__Gammaproteobacteria;o__Burkholderiales;f__Burkholderiaceae;g__Burkholderia-Caballeronia-Paraburkholderia;	0.00006	0.00009
OTU4813	p__Proteobacteria;c__Gammaproteobacteria;o__Enterobacterales;f__Enterobacteriaceae;g__Cedecea;	0.06298	0.00180
OTU13138	p__Proteobacteria;c__Gammaproteobacteria;o__Pseudomonadales;f__Moraxellaceae;g__Acinetobacter;s__Acinetobacter_johnsonii	0.00267	0.00001
OTU13132	p__Bacteroidota;c__Bacteroidia;o__Sphingobacteriales;f__Sphingobacteriaceae;g__Sphingobacterium;	0.07777	0.00161
OTU3026	p__Proteobacteria;c__Gammaproteobacteria;o__Pseudomonadales;f__Pseudomonadaceae;g__Pseudomonas;	0.15132	0.00013

OTU9424	p__Verrucomicrobiota;c__Verrucomicrobiae;o__Chthoniobacterales;f__Xiphinematobacteraceae;g__Candidatus_Xiphinematobacter;	0.00029	0.00491
OTU11067	p__Proteobacteria;c__Gammaproteobacteria;o__Pseudomonadales;f__Pseudomonadaceae;g__Pseudomonas;	0.15129	0.00443
OTU4980	p__Proteobacteria;c__Alphaproteobacteria;o__Rhizobiales;f__Hyphomicrobiaceae;g__Pedomicrobium;	0.00030	0.00309
OTU9582	p__Actinobacteriota;c__Actinobacteria;o__Corynebacteriales;f__Nocardiaceae;g__Rhodococcus;	0.00010	0.00006
OTU10785	p__Proteobacteria;c__Alphaproteobacteria;o__Rhizobiales;f__Rhizobiaceae;g__Allorhizobium-Neorhizobium-Pararhizobium-Rhizobium;	0.01463	0.00137
OTU13162	p__Proteobacteria;c__Alphaproteobacteria;o__Rickettsiales;f__Anaplasmataceae;g__Wolbachia;	0.00947	0.00000
OTU951	p__Proteobacteria;c__Alphaproteobacteria;o__Rhizobiales;f__Rhizobiaceae;g__Phyllobacterium;	0.00094	0.00148

2			
OTU4367	p__Proteobacteria;c__Alphaproteobacteria;o__Rhizobiales;f__Rhizobiaceae;g__Allorhizobium-Neorhizobium-Pararhizobium-Rhizobium;	0.00132	0.00014
OTU8670	p__Proteobacteria;c__Alphaproteobacteria;o__Sphingomonadales;f__Sphingomonadaceae;g__Sphingomonas;	0.00040	0.00509
OTU10769	p__Proteobacteria;c__Alphaproteobacteria;o__Rhizobiales;f__Rhizobiaceae;g__Phyllobacterium;	0.53597	0.00354
OTU13080	p__Proteobacteria;c__Gammaproteobacteria;o__Xanthomonadales;f__Rhodanobacteraceae;g__Luteibacter;	0.00372	0.00017
OTU9096	p__Actinobacteriota;c__Actinobacteria;o__Micrococcales;f__Micrococcaceae;g__Arthrobacter;	0.00076	0.00309
OTU8862	p__Actinobacteriota;c__Actinobacteria;o__Frankiales;f__Acidothermaceae;g__Acidothermus;	0.00015	0.00398

OTU9027	p__Actinobacteriota;c__Actinobacteria;o__Corynebacteriales;f__Mycobacteriaceae;g__Mycobacterium;	0.00898	0.01053
OTU10756	p__Proteobacteria;c__Alphaproteobacteria;o__Rhizobiales;f__Beijerinckiaceae;g__Microvirga;	0.00060	0.00206
OTU10754	p__Actinobacteriota;c__Actinobacteria;o__Corynebacteriales;f__Mycobacteriaceae;g__Mycobacterium;	0.00178	0.00046
OTU10759	p__Proteobacteria;c__Alphaproteobacteria;o__Rhizobiales;f__Devosiaceae;g__Devosia;	0.00180	0.00311
OTU5337	p__Proteobacteria;c__Gammaproteobacteria;o__Burkholderiales;f__Burkholderiaceae;g__Burkholderia-Caballeronia-Paraburkholderia;	0.00266	0.00085
OTU13123	p__Actinobacteriota;c__Actinobacteria;o__Micrococcales;f__Microbacteriaceae;g__Leucobacter;	0.00270	0.00117
OTU128	p__Proteobacteria;c__Alphaproteobacteria;o__Rhizobiales;f__Devosiaceae;g__Devosia;	0.00087	0.00008

70			
OTU8040	p__Proteobacteria;c__Alphaproteobacteria;o__Rhizobiales;f__Xanthobacteraceae;g__unclassified_f__Xanthobacteraceae;	0.00017	0.00424
OTU9114	p__Proteobacteria;c__Alphaproteobacteria;o__Sphingomonadales;f__Sphingomonadaceae;g__Sphingopyxis;	0.00068	0.00015
OTU9062	p__Proteobacteria;c__Alphaproteobacteria;o__Reyranellales;f__Reyranellaceae;g__Reyranella;	0.00082	0.00313
OTU9069	p__Actinobacteriota;c__Actinobacteria;o__Streptomycetales;f__Streptomycetaceae;g__Streptomyces;	0.00073	0.00655
OTU9029	p__Proteobacteria;c__Alphaproteobacteria;o__Rhizobiales;f__Xanthobacteraceae;g__unclassified_f__Xanthobacteraceae;	0.00206	0.01749
OTU10753	p__Proteobacteria;c__Alphaproteobacteria;o__Rhizobiales;f__Xanthobacteraceae;g__Bradyrhizobium;	0.00803	0.02904

OTU8157	p__Proteobacteria;c__Alphaproteobacteria;o__Sphingomonadales;f__Sphingomonadaceae;g__Ellin6055;	0.00128	0.00490
OTU2601	p__Proteobacteria;c__Alphaproteobacteria;o__Rhizobiales;f__Rhizobiaceae;g__Phyllobacterium;	0.00021	0.00000
OTU8129	p__Actinobacteriota;c__Actinobacteria;o__Streptomycetales;f__Streptomycetaceae;g__Streptomyces;	0.00026	0.00183
OTU13249	p__Actinobacteriota;c__Actinobacteria;o__Micrococcales;f__Microbacteriaceae;g__Microbacterium;	0.01932	0.00201
OTU7483	p__Actinobacteriota;c__Actinobacteria;o__Micrococcales;f__Microbacteriaceae;g__Plantibacter;	0.01012	0.00109
OTU10766	p__Proteobacteria;c__Gammaproteobacteria;o__Burkholderiales;f__Comamonadaceae;g__Variovorax;	0.03875	0.00323
OTU107	p__Bacteroidota;c__Bacteroidia;o__Sphingobacteriales;f__Sphingobacteriaceae;g__Pedobacter;	0.14361	0.00666

67			
OTU10764	p__Proteobacteria;c__Alphaproteobacteria;o__Rhizobiales;f__Rhizobiaceae;g__Mesorhizobium;	0.00336	0.00421
OTU10765	p__Proteobacteria;c__Gammaproteobacteria;o__Enterobacterales;f__unclassified_o__Enterobacterales;g__unclassified_o__Enterobacterales;	0.12929	0.00022
OTU10763	p__Proteobacteria;c__Gammaproteobacteria;o__Burkholderiales;f__Comamonadaceae;g__Delftia;	0.00320	0.00010
OTU10761	p__Proteobacteria;c__Alphaproteobacteria;o__Rhizobiales;f__Rhizobiaceae;g__Allorhizobium-Neorhizobium-Pararhizobium-Rhizobium;	0.01160	0.00137
OTU13140	p__Proteobacteria;c__Gammaproteobacteria;o__Pseudomonadales;f__Moraxellaceae;g__Acinetobacter;	0.00058	0.00000
OTU10768	p__Proteobacteria;c__Alphaproteobacteria;o__Rhizobiales;f__Rhizobiaceae;g__Mesorhizobium;	0.00584	0.00118

OTU10540	p__Proteobacteria;c__Alphaproteobacteria;o__Sphingomonadales;f__Sphingomonadaceae;g__Sphingomonas;	0.00024	0.00013
OTU4062	p__Proteobacteria;c__Alphaproteobacteria;o__Rhizobiales;f__Labraceae;g__Labrys;	0.00030	0.00014
OTU12315	p__Actinobacteriota;c__Thermoleophilia;o__Solirubrobacterales;f__67-14;g__unclassified_f__67-14;	0.00078	0.00553
OTU8054	p__Actinobacteriota;c__Thermoleophilia;o__Solirubrobacterales;f__Solirubrobacteraceae;g__Solirubrobacter;	0.00066	0.00739
OTU17780	p__Actinobacteriota;c__Actinobacteria;o__Corynebacteriales;f__Mycobacteriaceae;g__Mycobacterium;	0.00004	0.00119
OTU13101	p__Proteobacteria;c__Gammaproteobacteria;o__Burkholderiales;f__Comamonadaceae;g__Comamonas;	0.00040	0.00001
OTU953	p__Actinobacteriota;c__Actinobacteria;o__Corynebacteriales;f__Mycobacteriaceae;g__Mycobacterium;	0.00083	0.00739

2			
OTU9124	p__Proteobacteria;c__Alphaproteobacteria;o__Rhizobiales;f__Xanthobacteraceae;g__unclassified_f__Xanthobacteraceae;	0.00083	0.00771
OTU8236	p__Proteobacteria;c__Alphaproteobacteria;o__Rhizobiales;f__Methyloligellaceae;g__unclassified_f__Methyloligellaceae;	0.00043	0.00535
OTU9140	p__Actinobacteriota;c__Thermoleophilia;o__Solirubrobacterales;f__67-14;g__unclassified_f__67-14;	0.00035	0.00522

Note: p, phylum; c, class; o, order; f, family; g, genus; NJH, tissue sample of *C. militaris* in sclerotia. FZT, attached soil around the *C. militaris* 1 cm.

Table S2 Biochemical analysis and morphological characteristics of bacteria isolated from *C. militaris* sclerotia.

Test index	E28	J16	B21	B26	D4	E49	C1
Semi-solid agar	-	+	-	+	+	-	+
Ornithine decarboxylase broth	-	+	-	+	-	+	+
Lysine decarboxylase broth	-	+	-	+	-	-	+
Amino acid decarboxylase control	-	+	-	+	-	+	-
Simon's citrate	+	+	-	+	-	-	+
Hydrogen sulfide	-	+	-	+	-	-	-
Urease	+	+	+	+	+	-	+
Methyl Red Voges	-	+	-	-	-	-	-

Proskauer Broth							
Phenylalanine	-	-	-	-	-	-	-
Mannitol	+	-	-	+	-	-	-
Inositol	-	-	-	-	-	-	-
Sorbitol	-	-	-	-	-	-	-
Meliobiose	-	-	-	-	-	-	-
Ribohydrin	-	-	-	-	-	-	-
Raffinose	-	-	-	+	-	-	-
Gram positive test	G^+	G^-	G^-	G^-	G+	G^-	G^-
Bacterial size (μm)	1.16-1.56 × 0.76-0.80	1.6-1.76 × 0.51-0.71	1.52-3.12 × 0.98-1.16	0.87-1.33 × 0.44-0.47	2.14-3.13 × 1.07-1.25	0.94-1.12 × 0.85-0.89	0.80-1.12 × 0.54-0.63

Notes: E28, Rhodococcus jostii; J16, Achromobacter marplatensis; B21, Acinetobacter lwoffii; B26, Sphingobacterium multivorum; D4, Mycobacterium stephanolepidis; E49, Variovorax gossypii; C1, Pseudomonas protegens.

Table S3 Transcriptomic regulation of genes.

Gene_id	Gene name	NH_4 vs Peptone				NO_3 vs Peptone				NH_4 vs NO_3			
		FC	Log2FC	Padjust	Regulate	FC	Log2FC	Padjust	Regulate	FC	Log2FC	Padjust	Regulate
CCM_04436	cns1	——	——	——	——	4.254	2.089	2.11E-12	up	0.251	1.994	1.18E-28	up
CCM_04437	cns2	——	——	——	——	3.388	1.761	1.87E-17	up	0.433	1.208	1.84E-26	up
CCM_04438	cns3	——	——	——	——	——	——	——	——	——	——	——	——
CCM_00622	nuc5	——	——	——	——	——	——	——	——	——	——	——	——
CCM_05	——	——	——	——	——	——	——	——	——	——	——	——	——

789													
CCM_06768	——	2.798	1.485	9.55E-16	up	——	——	——	——	2.094	1.066	8.86E-12	down
CCM_07353	——	0.492	-1.023	0.001	down	3.765	1.913	7.94E-11	up	0.133	2.906	2.37E-54	up
CCM_05697	——	2.092	1.065	0.0001	up	4.089	2.032	8.40E-15	up	——	——	——	——
CCM_09444	——	2.852	1.512	6.80E-05	up	3.52	1.816	1.15E-06	up	——	——	——	——
CCM_02951	——	0.326	-1.618	2.73E-09	down	——	——	——	——	0.205	2.288	2.77E-38	up
CCM_02302	——	0.297	-1.7495	1.25E-05	down	——	——	——	——	0.241	-2.052	1.73E-21	down

Note: FC stands for Fold Change. “—”, indicates not shown in transcriptome data.

Table S4. Primers used for RT-qPCR.

Number	Name	Gene ID	DNA sequence （5’—3’）	Gene description
1	cns1	CCM_04436-F	TGACATTGTATGCGAGAA	Protein with dehydrogenase oxidoreductase domains
		CCM_04436-R	GAGCCTTGTGATTGATTG	
2	cns2	CCM_04437-F	AATGACAACAGCATCTTGATTC	Protein with metal-dependent phosphohydrolase domain
		CCM_04437-R	AGCAGCTCTACGAGAACT	
3	cns3	CCM_04438-F	ACCACCTACGAGATTCAG	hypothetical protein
		CCM_04438-R	CATCGCAGAGAAGATACG	
4	nuc5	CCM_00622-F	CAATGACCGAATGTGATG	5’-nucleotidase
		CCM_00622-R	CGCAACTGAAGGATAGAA	
5	_	CCM_05789-F	ATTGGTCGTAGTGTTGAA	adenylosuccinate lyase

		CCM_05789-R	CCTGTATTTCTGGATCTTTC	
6	_	CCM_06768-F	TGAGTATTACAGTCATTCT	adenylosuccinate synthetase
		CCM_06768-R	TATCGCTAATCTTTCCTT	
7	_	CCM_07353-F	CAGAAATTAAGATGCGGATG	adenylosuccinate synthetase
		CCM_07353-R	GATGCTGTCGTAGTTGTTA	
8	_	CCM_05697-F	AACGACAACCGCATTAAG	glutamine synthetase
		CCM_05697-R	AACTTCTTCTTGGAGATGAC	
9	_	CCM_09444-F	GGTATCCGTATTGTCAAGT	NAD-specific glutamate dehydrogenase
		CCM_09444-R	AAGACCGTAATTCTCATCAA	
10	_	CCM_02951-F	CAGTGTCAAGTGGCTTAA	nitrate reductase
		CCM_02951-R	CTCGGTTATCGTAGATGTG	
11	_	CCM_02302-F	ATACACCGTACCAAGCCCAA	hypothetical protein
		CCM_02302-R	TTTGTTACTGCCATGCCTCG	

12	18SrRNA	F	ATTTCCTTTCTGCGACTA	Internal reference gene
		R	GATGGTATGTTATTGCTGTA	

Table S5 RT-qPCR validation results.

NO.	Gene ID	$2^{-\Delta Ct}$ (NH_4vsPeptone)	$2^{-\Delta Ct}$ (NO_3vsPeptone)	$2^{-\Delta Ct}$ (NH_4vsNO_3)	Gene description
1	CCM_04436	3.84	5.00	0.71	cns1
2	CCM_04437	3.64	2.19	1.56	cns2
3	CCM_04438	5.72	6.97	0.76	cns3
4	CCM_00622	4.67	1.35	3.50	5'-nucleotidase
5	CCM_05789	0.81	1.29	0.66	adenylosuccinate lyase
6	CCM_06768	1.95	2.27	0.91	adenylosuccinate synthetase
7	CCM_07353	1.12	4.93	0.22	adenylosuccinate synthetase
8	CCM_07353	1.46	4.59	0.32	glutamine synthetase
9	CCM_09444	2.08	5.56	0.38	NAD-specific glutamate dehydrogenase
10	CCM_02951	0.29	1.23	0.26	nitrate reductase

11	CCM_02302	0.32	2.86	0.31	hypothetical protein

77 **Table S6** Abundance and predicted functions of 47 genera in sclerotia and in attached soil.

No.	Class	Genus	CMS	AS	Sclerotia/Soil	Predicted Function
1	High abundance (> 0.1%)	Rhodococcus	0.16690	0.00440	37.89	aromatic compound degradation;aromatic hydrocarbo degradation; chemoheterotrophy; hydrocarbon degradation;
2		Phyllobacterium	0.15649	0.00535	29.27	aerobic chemoheterotrophy; chemoheterotrophy
3		Pseudomonas	0.11969	0.00605	19.78	aerobic chemoheterotrophy; chemoheterotrophy
4		Pedobacter	0.04811	0.00666	7.22	aerobic chemoheterotrophy; chemoheterotrophy
5		Labrys	0.04609	0.00124	37.23	aerobic chemoheterotrophy;

						chemoheterotrophy
6		unclassified_Enterobacterales	0.04414	0.00022	201.31	None
7		Achromobacter	0.03568	0.00079	44.98	aerobic chemoheterotrophy; chemoheterotrophy;nitrate reduction; nitrate respiration; nitrogen respiration;
8		Ensifer	0.03512	0.00535	6.57	aerobic chemoheterotrophy; chemoheterotrophy; nitrate reduction;
9		Sphingobacterium	0.02303	0.00161	14.29	ureolysis
10		Cedecea	0.01904	0.00180	10.56	fermentation ; nitrate_reduction ; chemoheterotrophy；
11		Variovorax	0.01132	0.00323	3.50	None
12		Ochrobactrum	0.00935	0.00019	48.33	None

13		Allorhizobium-Neorhizobium-Pararhizobium-Rhizobium	0.00773	0.00288	2.68	aerobic chemoheterotrophy; chemoheterotrophy; nitrogen fixation;
14		Stenotrophomonas	0.00687	0.00138	4.99	aerobic chemoheterotrophy; chemoheterotrophy; nitrate reduction;nitrate respiration;nitrogen respiration; animal parasites or symbionts;
15		Plantibacter	0.00647	0.00109	5.94	None
16		Microbacterium	0.00569	0.00201	2.84	aerobic chemoheterotrophy; chemoheterotrophy;
17		Mycobacterium	0.00305	0.01958	0.16	aerobic chemoheterotrophy; chemoheterotrophy;
18		Wolbachia	0.00287	0.00000	0.00287	None

19		Mesorhizobium	0.00274	0.00537	0.51	aerobic chemoheterotrophy; chemoheterotrophy; ureolysis;
20		Bradyrhizobium	0.00271	0.02904	0.09	aerobic chemoheterotrophy; chemoheterotrophy; nitrogen fixation;
21		Bosea	0.00244	0.00096	2.54	aerobic chemoheterotrophy; chemoheterotrophy; dark oxidation of sulfur comounds
22		Inquilinus	0.00169	0.00019	8.86	None
23		Chryseobacterium	0.00148	0.00196	0.76	aerobic chemoheterotrophy; chemoheterotrophy;
24		Acinetobacter	0.00120	0.00001	123.67	aerobic chemoheterotrophy; chemoheterotrophy;
25		Luteibacter	0.00116	0.00017	6.90	None

26		Delftia	0.00103	0.00010	10.60	None
27		Burkholderia-Caballeronia-Paraburkholderia	0.00101	0.00094	1.07	animal_parasites_or_symbionts
28	Medium abundance	unclassified_Xanthobacteraceae	0.00085	0.02944	0.03	None
29		Leucobacter	0.00083	0.00117	0.71	aerobic_chemoheterotrophy ; chemoheterotrophy;
30		Devosia	0.00060	0.00320	0.19	aerobic chemoheterotrophy; chemoheterotrophy;
31		Rhodopseudomonas	0.00054	0.00015	3.50	photoheterotrophy ; phototrophy ; chemoheterotrophy;
32		Ellin6055	0.00049	0.00490	0.10	aerobic_chemoheterotrophy ; chemoheterotrophy;

33		Streptomyces	0.00031	0.01334	0.02	aerobic chemoheterotrophy; chemoheterotrophy;
34		Arthrobacter	0.00026	0.00309	0.09	None
35		Reyranella	0.00026	0.00313	0.08	aerobic chemoheterotrophy; chemoheterotrophy;
36		Sphingomonas	0.00025	0.00522	0.05	aerobic_chemoheterotrophy ; chemoheterotrophy;
37		unclassified_67-14	0.00024	0.01075	0.02	
38		Sphingopyxis	0.00021	0.00015	1.47	aerobic_chemoheterotrophy ; chemoheterotrophy
39		Solirubrobacter	0.00019	0.00739	0.03	aerobic_chemoheterotrophy ; chemoheterotrophy
40		unclassified_Methyloligellac	0.00014	0.00535	0.03	None

		eae				
41	Low abundance (< 0.01%)	Comamonas	0.00014	0.00001	14.33	aerobic chemoheterotrophy; chemoheterotrophy; hydrocarbon degradation; plastic degradation;
42		Microvirga	0.00014	0.00206	0.07	aerobic chemoheterotrophy; chemoheterotrophy;
43		Candidatus_Xiphinematobacter	0.00011	0.00491	0.02	animal parasites or symbionts
44		Pedomicrobium	0.00009	0.00309	0.03	aerobic chemoheterotrophy; chemoheterotrophy;
45		Acidothermus	0.00007	0.00398	0.02	aerobic_chemoheterotrophy ; chemoheterotrophy
46		unclassified_IMCC26256	0.00005	0.00364	0.02	None

47		unclassified_Ilumatobacteraceae	0.00005	0.00000	0.00005	None

79
